# Automated syndrome diagnosis by three-dimensional facial imaging

**DOI:** 10.1038/s41436-020-0845-y

**Published:** 2020-06-01

**Authors:** Benedikt Hallgrímsson, J. David Aponte, David C. Katz, Jordan J. Bannister, Sheri L. Riccardi, Nick Mahasuwan, Brenda L. McInnes, Tracey M. Ferrara, Danika M. Lipman, Amanda B. Neves, Jared A. J. Spitzmacher, Jacinda R. Larson, Gary A. Bellus, Anh M. Pham, Elias Aboujaoude, Timothy A. Benke, Kathryn C. Chatfield, Shanlee M. Davis, Ellen R. Elias, Robert W. Enzenauer, Brooke M. French, Laura L. Pickler, Joseph T. C. Shieh, Anne Slavotinek, A. Robertson Harrop, A. Micheil Innes, Shawn E. McCandless, Emily A. McCourt, Naomi J. L. Meeks, Nicole R. Tartaglia, Anne C.-H. Tsai, J. Patrick H. Wyse, Jonathan A. Bernstein, Pedro A. Sanchez-Lara, Nils D. Forkert, Francois P. Bernier, Richard A. Spritz, Ophir D. Klein

**Affiliations:** 1 Department of Cell Biology & Anatomy, Alberta Children’s Hospital Research Institute and McCaig Bone and Joint Institute, Cumming School of Medicine, University of Calgary, Calgary, AB Canada; 2 Biomedical Engineering Graduate Program, University of Calgary, Calgary, AB Canada; 3 Human Medical Genetics and Genomics Program and Department of Pediatrics, University of Colorado School of Medicine, Aurora, CO USA; 4 Program in Craniofacial Biology and Department of Orofacial Sciences, University of California, San Francisco, CA USA; 5 Department of Medical Genetics, Alberta Children’s Hospital Research Institute, Cumming School of Medicine, University of Calgary, Calgary, AB Canada; 6 Department of Pediatrics, University of Colorado School of Medicine, Aurora, CO USA; 7 Department of Pediatrics, Cedars Sinai Medical Center & David Geffen School of Medicine at UCLA, Los Angeles, CA USA; 8 Department of Psychiatry and Behavioral Sciences, Stanford University, Stanford, CA USA; 9 Department of Pediatric Ophthalmology, University of Colorado School of Medicine, Aurora, CO USA; 10 Department of Surgery, Division of Plastic and Reconstructive Surgery, University of Colorado School of Medicine, Aurora, CO USA; 11 Department of Pediatrics and Institute for Human Genetics, University of California, San Francisco, CA USA; 12 Department of Surgery, Alberta Children’s Hospital Research Institute, Cumming School of Medicine, University of Calgary, Calgary, AB Canada; 13 Division of Ophthalmology, Department of Surgery & Department of Medical Genetics, Cummings School of Medicine, University of Calgary, Calgary, AB Canada; 14 Department of Pediatrics, Stanford School of Medicine, Stanford, CA USA; 15 Department of Radiology, Alberta Children’s Hospital Research Institute, and Hotchkiss Brain Institute, Cumming School of Medicine, University of Calgary, Calgary, AB Canada; 16 Present Address: Department of Pediatrics, Geisinger Medical Center, Danville, PA USA

**Keywords:** syndromes, facial imaging, deep phenotyping, diagnosis, morphometrics

## Abstract

**Purpose:**

Deep phenotyping is an emerging trend in precision medicine for genetic disease. The shape of the face is affected in 30–40% of known genetic syndromes. Here, we determine whether syndromes can be diagnosed from 3D images of human faces.

**Methods:**

We analyzed variation in three-dimensional (3D) facial images of 7057 subjects: 3327 with 396 different syndromes, 727 of their relatives, and 3003 unrelated, unaffected subjects. We developed and tested machine learning and parametric approaches to automated syndrome diagnosis using 3D facial images.

**Results:**

Unrelated, unaffected subjects were correctly classified with 96% accuracy. Considering both syndromic and unrelated, unaffected subjects together, balanced accuracy was 73% and mean sensitivity 49%. Excluding unrelated, unaffected subjects substantially improved both balanced accuracy (78.1%) and sensitivity (56.9%) of syndrome diagnosis. The best predictors of classification accuracy were phenotypic severity and facial distinctiveness of syndromes. Surprisingly, unaffected relatives of syndromic subjects were frequently classified as syndromic, often to the syndrome of their affected relative.

**Conclusion:**

Deep phenotyping by quantitative 3D facial imaging has considerable potential to facilitate syndrome diagnosis. Furthermore, 3D facial imaging of “unaffected” relatives may identify unrecognized cases or may reveal novel examples of semidominant inheritance.

## INTRODUCTION

Of >7000 rare syndromes in humans, 30–40% involve dysmorphic craniofacial features^1^ and such features often contribute to initial clinical diagnoses. Diagnoses enable affected individuals and their families to access resources, prognoses, and available treatments. However, access to medical genetics remains limited, especially outside of the developed world. Increasingly, expert systems have been deployed to assist syndrome diagnosis, including computer databases^2^ and analytic software,^3^ as well as human expert^4^ and online services.^5^ In parallel, diagnosis has been greatly facilitated by improvements to molecular diagnostic testing and sequencing.^6^ Nevertheless, testing is expensive and access remains limited outside high-income countries.^7^ Even with sequencing, nearly 50% of all patients remain undiagnosed.^8^ For these reasons, as well as the emerging importance of telemedicine, improvements in clinical decision support systems via automated dysmorphology assessment are beneficial.

Previous work has addressed the use of standard two-dimensional (2D) facial images for syndrome diagnosis.^1,5,9^ However, three-dimensional (3D) facial images contain more shape information than corresponding 2D images. Further, 3D photogrammetry is not affected by focal depth, which can produce significant distortion of apparent morphology in 2D images.^10^ Decreasing cost of 3D cameras along with advances in computing and image analysis^11^ have facilitated access to 3D facial photogrammetry; indeed, consumer level, smartphone-based 3D cameras are already nearly capable of supporting clinical 3D morphometrics (Fig. S1). 3D photogrammetry has been used as an approach to deep phenotyping of individual genetic syndromes with facial dysmorphology,^12^ and is widely used in plastic surgery, dermatology, and orthodontics.^13^ However, 3D facial imaging has not yet been developed as a tool for automated diagnosis of dysmorphic syndromes.

We evaluated 3D facial photogrammetry as a novel expert system for automated diagnosis of facial dysmorphic syndromes. Under the auspices of the National Institute for Dental and Craniofacial Research (NIDCR) FaceBase initiative (https://www.facebase.org/), we assembled a “library” that currently contains 3D facial images from over 5900 individuals with syndromes with facial dysmorphism, as well as over 900 unaffected relatives. This library is available through FaceBase. We evaluated facial shape in a data freeze that includes 3D images from 3327 individuals with 396 different syndromes, and 727 unaffected relatives. For most analyses, we also incorporated a sample of 3003 unrelated, unaffected individuals. We quantified overall patterns of facial shape variation in syndromic and unaffected subjects and evaluated the accuracy of facial shape for classifying subjects to syndromes. We found that 3D facial shape correlates of syndromes account for a significant fraction of facial shape variation. Most syndromes are classifiable from facial shape with moderate-to-high accuracy, providing a rigorous quantitative framework for developing 3D facial photogrammetry as an expert system for syndrome diagnosis.

## MATERIALS AND METHODS

### Study characteristics and demographics

**Fig. 1:**
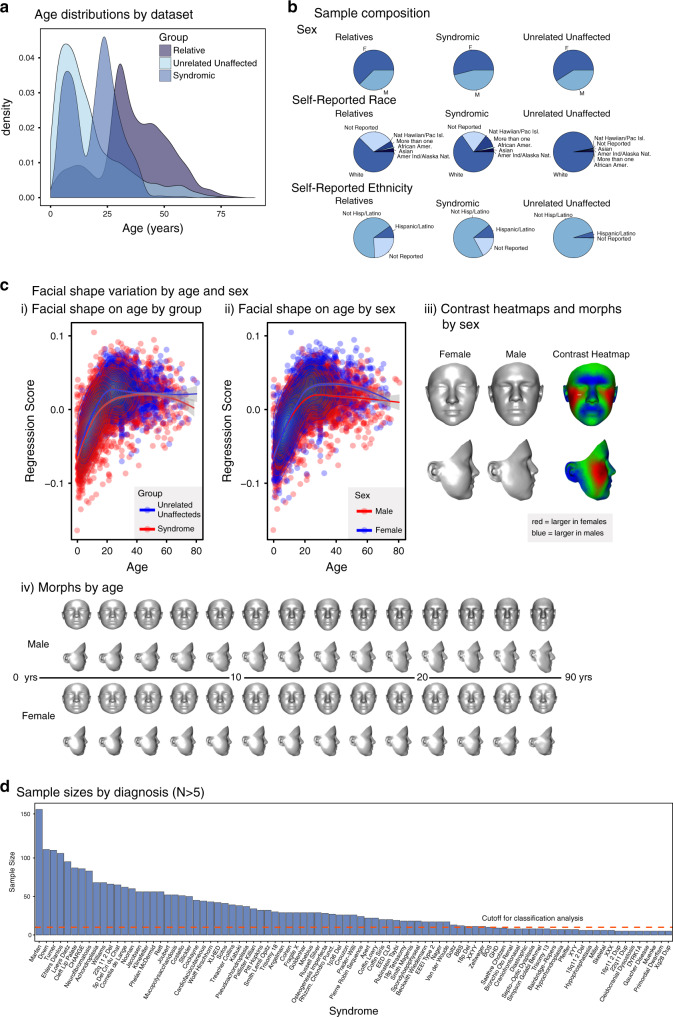
Composition of the 3D facial image library. (**a**) Age distribution for syndromic; unrelated, unaffected; and unaffected relative subjects. (**b**) Polynomial age regression score against age plotted by group (syndromic versus unrelated, unaffected) (**i**) and sex (**ii**). 3D heatmaps showing regions of facial shape differences between sexes (**iii**). Shape morphs showing average facial shape changes with age by sex (**iv**). (**c**) Sample composition by self-reported sex, ethnicity, and race, as specified in the National Institutes of Health (NIH) reporting guidelines (NOT-OD-15–089). (**d**) Distribution of sample sizes by syndrome for all syndromes with *n* > 5. The dotted red line shows the cut-off for inclusion in the classification analysis at *n* ≥ 10).

From 2013 through 2019, we enrolled subjects at outpatient clinics and patient group meetings in the United States, Canada, and the United Kingdom (Table S1). Inclusion criteria included diagnosis with a syndrome with known or possible effects on facial morphology. When possible, subjects’ relatives were enrolled. Subjects or their parents consented according to institutional review board (IRB) protocols of each center. The analysis is based on a data freeze of 3327 subjects with 396 syndromes (File S1), 727 of their apparently unaffected relatives, and 3003 unrelated, unaffected individuals, including 2851 from the facial shape genome-wide association study (GWAS) cohort of Shaffer et al.^14^ plus 152 enrolled through this project. Of syndromic subjects, 1555 had a molecular diagnosis and 1772 had only a clinical diagnosis. Subjects ranged in age from newborn to >80 years (Fig. 1a), with slightly more females than males (Fig. 1b). Self-reported race was predominantly white (83.1%) for the syndromic subjects (and almost exclusively so for the unrelated, unaffected subjects) and ethnicity was 87.3% non-Hispanic (Fig. 1b, Table S2), reflecting composition of patient meetings and clinic site populations. All study subjects or their parents provided written consent for sharing of recognizable facial images and relevant clinical data with qualified investigators approved by the National Institute of Dental and Craniofacial Research (NIDCR) Data Access Committee.

### Collection and curation of metadata

We obtained age, height, weight, head circumference, and relevant clinical data. Self-reported race and ethnicity were defined according to National Institute of Health (NIH) guidelines (NOT-OD-15–089). At patient meetings, syndrome diagnoses were self-reported. Genetic test records were obtained and reviewed when possible. At clinics, diagnoses were determined by a medical geneticist and molecular results were obtained when available. Provisional clinical diagnoses were amended based on follow-up clinical or test data.

### 3D facial imaging

We obtained 3D photogrammetric images of the face for all subjects. For 436 subjects, we used a Creaform Gemini camera. The remaining subjects were imaged with a 3dMDface camera system. Camera effects on classification accuracy accounted for only 0.3% of variation in facial shape, and classification results were identical with and without correction for camera effect. Subjects were imaged seated in a chair or a parent’s lap. Images were cropped to remove potentially confounding artifacts.

### 3D facial morphometric phenotyping

Morphometric phenotyping utilized a variation of our automated landmarking method.^15^ An average facial atlas was registered nonlinearly to each facial scan. To create the atlas, we selected a single scan from the unrelated, unaffected subject image set that was then cropped and decimated to ~2500 points, which represents a compromise between resolution and computational cost. The mesh was then registered to ten random scans from the unrelated, unaffected subject image set using the Optimal Step Non-Rigid Iterative Closest Point (N-ICP) algorithm.^16^ We then registered this average atlas to each scan using the same algorithm.

Since only a single atlas is landmarked, any number of landmarks can be obtained up to the resolution of the scan. However, increasing landmarks produces diminishing returns as neighboring landmarks tend to be correlated. For example, decomposition of our 2500 dense facial meshes produced fewer than 300 nonzero eigenvalues. An additional statistical issue arises when the number of coordinates (*p*) exceeds the number of observations (*n*).^17^ To optimize the tradeoff between the risk of overfitting and capturing relevant variation, we used 65 3D landmarks (Fig. S2).

### Statistical analyses

We analyzed the landmark data with geometric morphometrics in R.^18^ As described previously,^19^ we detected outliers using the Procrustes distance to the mean and the within-individual variance of the deviations from the average position of each landmark, and optimized the outlier threshold by running the classification analysis at different thresholds. We used principal components analysis (PCA), linear models implemented for landmark data,^20,21^ and canonical variates analysis (CVA) in geomorph,^22^ Morpho,^23^ shapes,^24^ Evomorph,^25^ and various custom functions in R.^26,27^

To standardize facial shape by age and sex, we evaluated the residuals of a linear model that included a three-term polynomial age predictor and sex (Fig. 1c).^21^ The variation attributable to these and other factors of interest were quantified with Procrustes multivariate analysis of variance (MANOVA), implemented in geomorph.^22^ To obtain unbiased estimate of sums of squares, we iterated across all combinations of the ordering of the terms in the model using type 1 sums of squares. All classification analyses were based on age and sex standardized data.

We used both the symmetric and asymmetric components of facial shape variation.^28^ Though facial asymmetry is a feature of some syndromes, the symmetrized data substantially outperformed either the unsymmetrized or the combined symmetric and asymmetric components (Fig. S3).

We quantified shape distances using the Procrustes distance. Integratedness was measured as the scaled variance of eigenvalues.^29^ Phenotypic severity is the average shape distance between the subjects with a syndrome and the mean for unaffected, unrelated subjects. Phenotypic distinctiveness is the shape distance between a syndrome and the nearest other syndrome in the data set. Finally, covariance distance measures the differences in the within-syndrome variances and covariances of traits (landmarks).

### Syndrome classification

To classify faces, we used both parametric (CVA) and machine learning methods. We tested various machine learning approaches, including deep neural networks, random forests, partial least squares, *k*-nearest neighbors, and high-dimensional regularized discriminant analysis models (HDRDA). Of these, HDRDA performed best (Fig. S4). HDRDA modifies linear discriminant analysis by allowing the sample covariance matrix to influence the within-class covariance matrix estimate with a pooling parameter, simultaneously shrinking the within-class covariance matrix toward the identity matrix with a regularization parameter.^30^ This allows the number of features (*p*) to exceed the number of individuals (*n*).^31^

We used a minimum syndrome sample size of 10 as a compromise between per-syndrome sample sizes and maximizing the number of syndromes included. We used a family level leave-one-out cross-validation (LOOCV). We also employed a 20-fold cross-validation strategy for comparison. However, *k*-fold cross-validation can underestimate performance for small samples, particularly if variation within syndromes is not normally distributed (File S2). This leads to underestimated sensitivities for syndromes with small *n* (Fig. S5). For syndromes with larger *n*, the two cross-validation approaches perform similarly.

For each subject, classification returns a vector of posterior probabilities for each class based on naïve priors. In this case, the naïve prior is the proportion of subjects belonging to that class. Thus, all subjects have an a priori 52.3% probability of being diagnosed as unaffected because that class comprises 52.3% of the data set. We used the posterior probabilities to obtain top-1, -3, and -10 classification results. Our analysis reports sensitivity (the proportion of subjects correctly classified), specificity (the proportion of subjects correctly identified as not having that syndrome), and balanced accuracy (the average of sensitivity and specificity).^32^

To analyze the classification of unaffected relatives, we used the set of families with at least one syndromic subject with a syndrome represented by *n* ≥ 10 and one unaffected relative facial scan (*n* = 479). We then fit the HDRDA model, iteratively leaving out the syndromic members of each family. No relatives were used in the training data for the model. The HDRDA model was trained on the full classification sample that included both syndromic subjects and unrelated, unaffected subjects.

## RESULTS

### Variation in facial shape

To quantify facial shape variation due to age, sex, and race, we modeled facial shape variation in the total syndromic sample and the unrelated, unaffected sample with polynomial predictors for age, sex, and race (Table S3). Age accounted for 14.4% of variance for syndromic subjects and 25.7% of variance among unrelated, unaffected subjects (Fig. 1c). The smaller variance attributable to age in syndromic subjects reflects the higher overall variance in this group. Sex accounted for less than 1% of variance for syndromic subjects and 2% for unaffected subjects. Self-reported race accounted for less than 2% of shape variance in both groups.

To quantify syndrome-related variation, we first standardized facial shape by age and sex. We performed this analysis for both the symmetric component of variation and the unsymmetrized (full Procrustes) data. When only the syndromic individuals were analyzed, syndrome diagnosis accounted for 14–15% of the total variation in facial shape, regardless of whether asymmetric facial variation was considered, and nearly 19% of the total variance when unrelated unaffected subjects were included (Table S4) (MANOVA, *p* < 0.001). This shows that syndrome diagnosis accounts for a considerable proportion of facial shape variation.

**Fig. 2:**
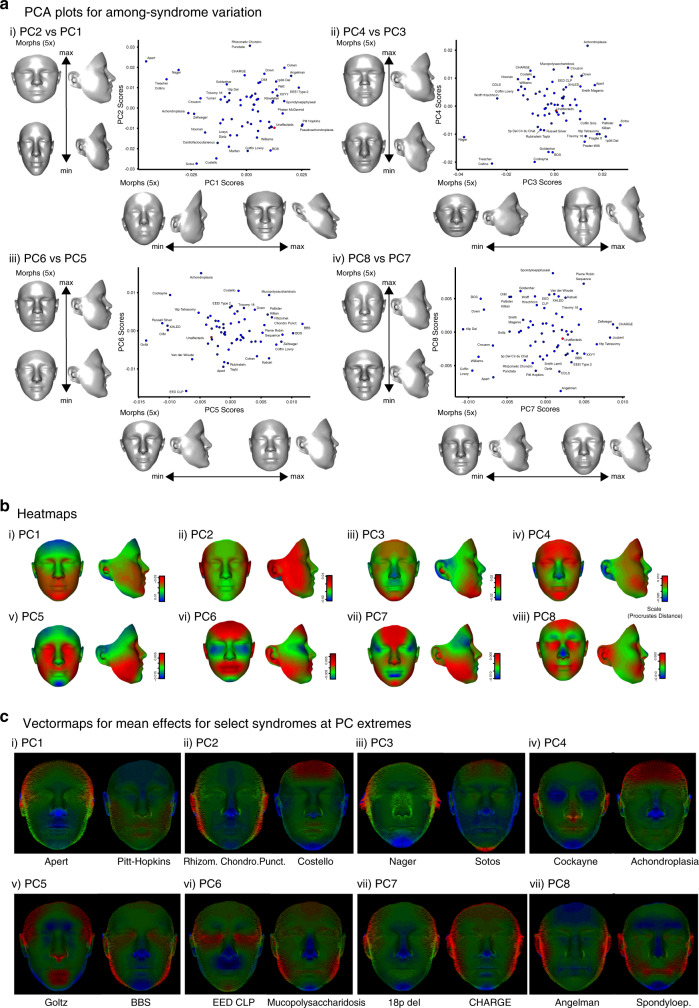
Principal components analysis (PCA) of the among-syndrome means. Each syndrome is represented by the average facial shape for that syndrome after regressing shape on polynomial age and sex. (**a**) Plots show the facial shape changes associated with each PC, scaled to 5 times the standard deviation of PC scores. (**b**) Heatmaps showing the regions of the face that vary most along each PC (red = larger, blue = smaller). (**c**) Vectormaps for syndromes that define the extremes of the PCA for the syndromic means. These are similar but not identical to the heatmaps in (**b**) because a syndromic mean can differ from the grand mean along multiple PCs. Both heatmaps and vectormaps are based on the distances between average meshes, registered in Procrustes space.

To visualize syndrome-related facial shape variation, we performed a PCA on the mean facial shape effects by syndrome. PCs 1–8 captured 60% of the resulting variation (Fig. 2). Syndromes that fall on extremes of the axes of variation captured by these PCs are similar to the PC morphs (Fig. 2c). We also provide mean shapes and heatmaps by syndrome (Fig. S6) as well as animations for these visualizations (Supplementary Videos 1–8). These results show the wide extent and qualitative nature of the variation in facial shape associated with syndrome diagnoses. As an adjunct to this paper, we present an online tool for visualization of all possible pairs of syndromes, including unaffecteds (https://genopheno.ucalgary.ca/Syndrome_gestalts/).

### Automated subject classification

**Fig. 3:**
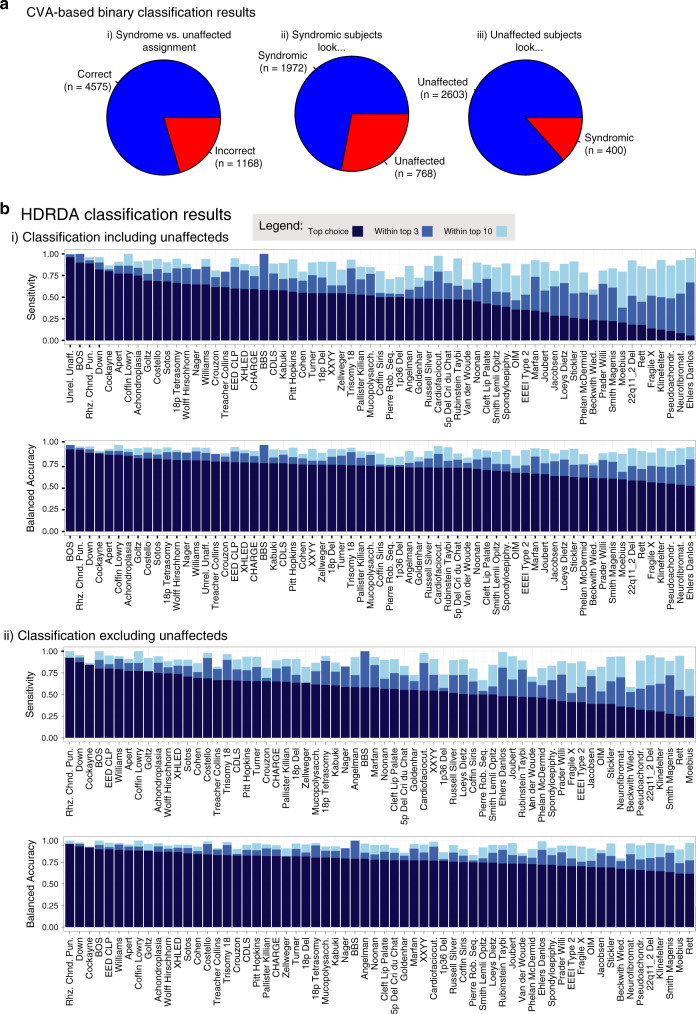
Syndrome classification. (**a**) Sensitivities for a two-group classification, syndromic versus unrelated, unaffected: (**i**) overall sensitivity; (**ii**) sensitivity for the syndromic subjects; (**iii**) sensitivity for unrelated, unaffected subjects. (**b**) Sensitivity and balanced accuracy (high-dimensional regularized discriminant analysis [HDRDA]). Top-1, -3, and -10 sensitivity and balanced accuracy by syndrome for the full classification sample that included both syndromic subjects and unrelated, unaffected subjects (**i**) and the syndrome-only classification sample (**ii**). Balanced classification accuracy by syndrome. Red lines depict grand mean top-1, -3, and -10 sensitivities and balanced accuracies.

We undertook a series of classification analyses based on the age and sex standardized facial shape data. We first considered the extent to which a common axis of difference distinguishes syndromic from unaffected facial shape by assigning syndromic subjects to a single class and using CVA to discriminate that class from unrelated, unaffected subjects. CVA correctly classified 2603 of 3003 (86.7%) unrelated, unaffected subjects and 1972 of 2736 (72%) syndromic subjects (Fig. 3a). Thus, even without regard to specific syndrome, 80% of study subjects could be correctly classified as either unaffected or syndromic based solely on facial shape.

To classify subjects to specific syndromes, we used HDRDA as well as CVA with and without the unrelated, unaffected group. When unaffected subjects were included, 96% were correctly classified as unaffected using HDRDA while 48.8% of syndromic subjects were correctly diagnosed to the correct syndrome (Fig. 3Bi). The overall classification rate to the correct syndrome was 71.8% and the correct diagnosis was listed among the top ten ranked diagnoses for 87.2% of syndromic subjects (Table S5). There was considerable variation in classification performance across diagnoses (Fig. 3Bi).

Most syndromic subjects for whom the correct syndrome was not the top choice were misclassified as unaffected, whereas unaffected, unrelated subjects were rarely misclassified as having a syndrome (Table S6). Accordingly, specificity for subjects correctly classified as not having a syndrome was over 99% for all syndromes. While syndromic subjects were occasionally classified to the wrong syndrome, no single syndrome received many misdiagnoses. Accordingly, specificity was only 67.3% for unaffected, unrelated subjects, reflecting the tendency for misclassified syndromic subjects to be classified as unrelated, unaffected.

As the number of subjects varied by group, it is useful to quantify overall classification performance by balanced accuracy, a metric that encompasses both true positive rate and true negative rate information (sensitivity and specificity).^32^ When the HDRDA analysis included unaffecteds, balanced accuracies ranged from a high of 95% for Bohring–Opitz syndrome (BOS) to a low of 53% for Ehlers–Danlos syndrome (Fig. 3Bii), a diagnostic category with many subtypes that were not distinguished here. When the HDRDA classification task excluded unaffecteds, the overall correct classification rate declined to 57.2%. However, the classification rate for syndromic subjects rose to 57.2%, sensitivity improved to 56.9%, and balanced accuracy to 78.1%. This is because “unaffected subject” was the most common misdiagnosis for syndromic subjects when that option was available. Balanced accuracies improved moderately as well (Fig. 3Bii). The full set of classification parameters are provided in Table S6.

CVA based classification performed less well, identifying the correct syndrome only 30% of the time when unaffecteds were included (Figure S7). HDRDA generally outperformed CVA in syndrome diagnosis. The full set of HDRDA posterior classification probabilities is shown in Figure S8.

### Determinants of classification performance

**Fig. 4:**
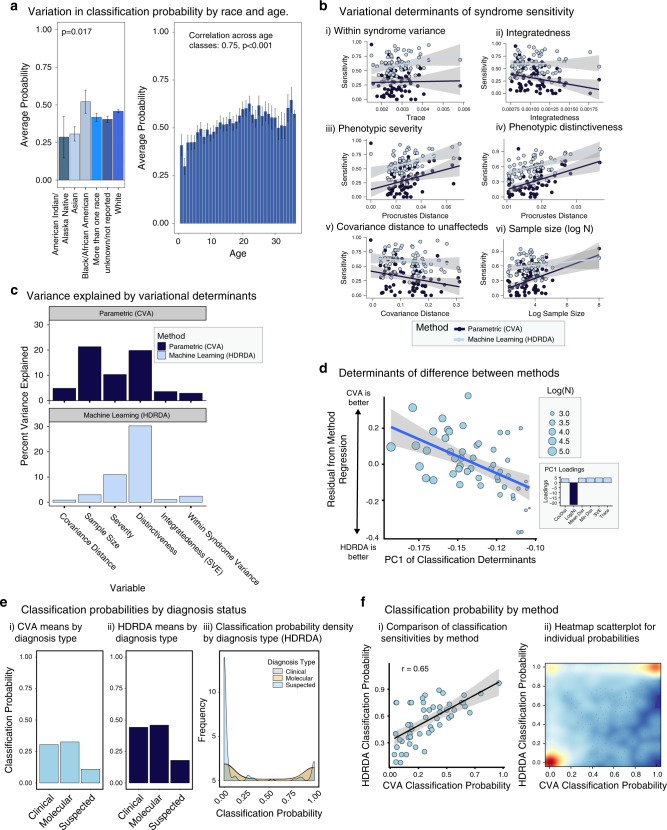
Determinants of sensitivity (high-dimensional regularized discriminant analysis [HDRDA] and canonical variates analysis [CVA]). (**a**) Classification accuracies plotted against potential determinants of classification accuracy. (**b**) Variation in classification accuracy attributable to potential determinants. (**c**) PC1 of classification determinants (accounting for 90% of variation) plotted against differences in performance between HDRDA and CVA. (**d**) Residual of regression for syndrome sensitivities for the two methods plotted against the first PC for the determinants of classification accuracy. (**e**) Classification probability as a function of diagnosis status. (**f**) By-syndrome sensitivity comparison for HDRDA and CVA classification.

To investigate determinants of classification, we examined the role of biological factors such as age, sex, ethnicity, and race as well as variational and sampling. Classification probability is similar by sex but correlates positively with age (*r* = 0.75, *p* < 0.001). Classification probabilities varied with race, with highest performance for Black/African American subjects and lowest for Asian subjects (Fig. 4a). Ethnicity was not a significant determinant.

We also examined the impact of variability, phenotypic severity, phenotypic distinctiveness, and integratedness of each syndrome, as well as diagnostic certainty for syndromic subjects. For the HDRDA analyses, only phenotypic severity and distinctiveness were associated with classification accuracy (*p* < 0.05) (Fig. 4b). For CVA, all factors except within-syndrome variance were significant. Combining all factors into a single model revealed that phenotypic distinctiveness accounted for the greatest variation in classification accuracy for both methods (Fig. 4c), while phenotypic severity was the second most important factor. Phenotypic severity may not be the major driver of classification performance because more severe syndromes tend to be more variable (*r* = 0.66, *p* < 0.001), meaning some of the gain in accuracy from increased severity is offset by increased variance.

Surprisingly, syndromes are not particularly likely to be confused with their nearest neighbors in shape space (Table S7). This suggests the number and composition of the syndromes included in the classification is important. Accordingly, we performed 250 parametric classification permutations with cross-validation, varying the number of syndromes from 2 to 50. In each permutation, the syndromes were chosen at random. This simulation showed that classification became less accurate as more syndromes were considered (Fig. S9).

Sample size influenced classification accuracy for CVA but not for HDRDA. Accordingly, differences in sensitivity between CVA and HDRDA were largely due to variation in sample size (Fig. 4c, d). Sensitivity for large-sample syndromes tended to be higher for CVA whereas sensitivity for small-sample syndromes was higher for HDRDA.

We also compared subjects with molecularly confirmed diagnosis to those with definitive clinical diagnoses and clinically suspected diagnoses. Molecularly confirmed subjects had higher classification probabilities than those with suspected diagnoses (Fig. 4Ei and ii), but classification probabilities for clinical and molecular diagnoses were similar (Fig. 4Eii–iii). Many individuals with suspected diagnoses had very low posterior probability values. Possibly, some suspected diagnoses were wrong and such individuals may have syndromes not included in the training set, effectively making them unclassifiable.

To determine the impact of classification method, we compared syndrome classification sensitivities and individual classification probabilities. HDRDA and CVA classification probabilities correlate, though for most syndromes HDRDA sensitivity was higher (*r* = 0.65, *p* < 0.001), suggesting rough agreement between methods (Fig. 4f). Examination of individual posterior probabilities showed that most individuals fall at either 0 or 1 for both methods (Fig. 4f). However, whereas many individuals were classified correctly with HDRDA but not with CVA, the opposite was rarely true.

### Unaffected relatives of syndromic subjects are disproportionately misclassified as syndromic

Finally, we evaluated the classification of unaffected relatives of syndromic subjects. We classified each unaffected relative as syndromic versus unaffected after training using HDRDA on the full syndromic sample in which relatives were not included. Our null hypothesis was that unaffected relatives of syndromic subjects would classify as unaffected at the same rate as unrelated, unaffected subjects.

**Fig. 5:**
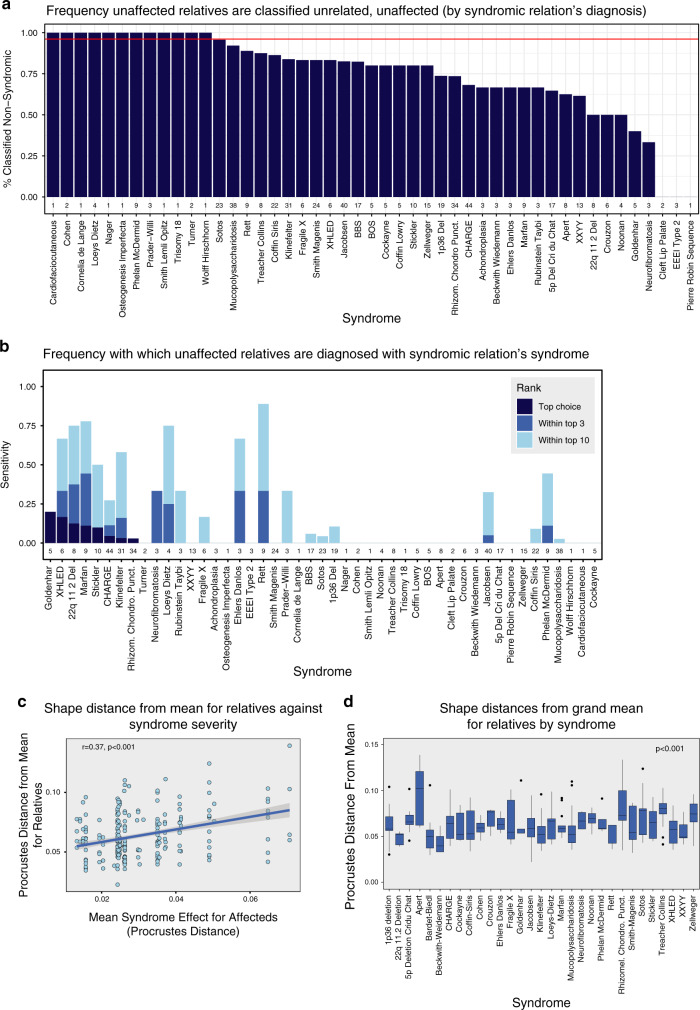
Diagnosis of unaffected relatives. (**a**) Sensitivities for unaffected relatives, grouped according to the diagnosis of the syndromic relation. (**b**) Frequency with which a syndromic subject’s diagnosis is also among the top-10 ranked diagnoses for the unaffected relative. (**c**) Phenotypic extremeness for relatives against the phenotypic severity of their relative’s syndrome. (**d**) Varation in phenotypic extremeness of relatives by syndrome.

Surprisingly, we found that relatives were significantly less likely to classify as unaffected compared with subjects in the unrelated, unaffected group (Chi-square [χ^2^] = 243.36, *p* < 2.2e-16). Relatives classified as unaffected only 77% of the time, in contrast to 96.1% for the unrelated, unaffected subjects (Fig. 5a). Even more intriguing were the patterns of apparent misdiagnosis. While the frequency of misdiagnosis varied by syndrome of the affected relative (Fig. 5b), HDRDA often diagnosed an unaffected relative to the same syndrome as their syndromic relative (Fig. 5b). This suggests that some of putatively unaffected relatives might, in fact, be affected. To investigate further, we considered whether unaffected relatives of subjects with more severe syndromes were more likely to differ from the grand mean. That turned out to be the case; in 332 unaffected relatives of syndromic subjects from 31 syndromes, the shape distance of each relative from the mean varied among syndromes (Fig. 5c, d) (Levene’s test for Procrustes distance, df = 30, F = 4.5, *p* < 0.0001). Furthermore, the extent of this shape effect in relatives was positively correlated with the phenotypic severity of the syndrome of their affected family member (linear model, MS = 0.008, F = 53.7). These results suggest that some unaffected relatives represent undiagnosed or incompletely penetrant syndromic cases.

## DISCUSSION

Human facial shape is highly polygenic,^14,19^ while most syndromes involve mutations in single genes. To investigate facial correlates of syndromes and facilitate development of automated diagnostic systems, we assembled a large library of 3D facial images of subjects with facial dysmorphism syndromes, as well as unaffected relatives. Potential uses include studies of within-syndrome heterogeneity, genotype–phenotype correlations, and comparison with animal models.

We analyzed 3D images and metadata from a data freeze of 3327 subjects with 396 different syndromes, 727 of their unaffected relatives, and 3003 unrelated, unaffected subjects. We used machine learning (HDRDA) and parametric (CVA) classification to evaluate the utility of 3D facial shape data for syndrome differential diagnoses, focusing on 64 syndromes with sample size *n* ≥ 10. Classification performance was superior by HDRDA, driven by superior performance for syndromes with small sample sizes. CVA is problematic for syndromes when sample size is smaller than the statistical degrees of freedom,^18^ whereas HDRDA is remarkably robust to sample size. This is important for rare syndromes.

The most important determinant of classification performance was distinctiveness of its facial phenotype—its nonproximity in shape space to other syndromes. This was more important than severity of the phenotype—the distance to the facial shape of unrelated, unaffected individuals. Phenotypically severe syndromes may be difficult to classify if other syndromes have similar phenotypes. For example, sensitivity for Treacher Collins syndrome is likely depressed because Nager syndrome often presents similar facial findings.^33^ As more syndromes are considered, classification becomes less accurate because of the increased chance of confusion among syndromes with similar effects. This complicates comparisons of classification studies that likely have different subject compositions.

Unexpectedly, within-syndrome variance—the range of variation of facial shape among individuals with the same syndrome—did not determine classification accuracy. This may reflect counteracting effects of syndrome severity and within-syndrome variance, as syndromes with more severe facial shape effects tend to be more variable.

Importantly, subjects with clinical but not molecular diagnoses were classified with accuracies similar to those with molecular confirmation, providing an important validation of the method. Subjects with merely suspected diagnoses, however, were classified with much lower accuracy. This may reflect incorrect clinical diagnoses of such individuals or atypical manifestations of a syndrome. Alternatively, individuals with suspected diagnoses may have a syndrome not included in the training set, making them effectively “unclassifiable” within our study. Further analysis will determine if classification probability profiles are informative. It is possible that the correct syndromes for such “unclassifiable” subjects have phenotypic features similar to those of diagnoses that are assigned the highest probabilities.

Strikingly, syndromic subjects’ unaffected relatives differed in important respects from unaffected, unrelated subjects. Relatives had greater tendency to depart from the mean facial shape for unrelated, unaffected subjects and were also much more likely to be misdiagnosed as syndromic, often to the same syndrome as their affected relative. These findings suggest that some relatives thought to be “unaffected” may in fact be exhibiting clinically mild manifestations of the same syndrome (*forme fruste*). Alternatively, some relatives of patients with recessive syndromes may manifest mild heterozygote effects. Larger samples of “unaffected” relatives per syndrome are needed to fully disentangle the causes of this phenomenon.

Race and ethnicity account for only a small proportion of facial shape variation, which is consistent with prior work.^34^ Nevertheless, our study overrepresents (83.1%) subjects who self-identified as white compared with the US population (76%), while subjects identifying as Black/African American, Asian, or American Indian/Alaska Native are underrepresented, as are Hispanic/Latino subjects to a lesser degree. Investigating possible bias in syndrome classification due to race and ethnicity is an important direction for future work. Facial imaging for syndrome diagnosis also has implications for privacy and ethics. The ability to infer medical information from faces may contribute to growing end-of-privacy fears and has potential psychological impacts that warrant attention.^35^

There are several previous approaches to syndrome classification from facial images, both 2D photographs of faces^9,36,37^ and using 3D photogrammetry.^38–40^ Gurovich et al. reported a sensitivity of 61% for classification of 2D images using a deep learning convolutional neural network method.^5^ While this is higher than the 48% sensitivity achieved here, direct comparison is difficult. Differences in syndrome composition of the classification task as well as the distribution of individuals across the included syndromes can dramatically affect overall classification accuracy. It is also likely that syndromes vary in how well they can be classified from 2D photographs versus 3D facial scans, depending on their specific phenotypic effects.

More important, 2D photographs and 3D facial scans contain different intrinsic information. Three-dimensional shape produces indirect variation (e.g., shadows) on a 2D photograph, whereas shape is quantified directly from a 3D mesh. Though 2D photographs are easier to obtain, 3D images are much less affected by camera angle, focal depth, and lighting. Counterintuitively, 2D data images are more complex than 3D meshes. The full dimensionality of color images is high and variation in this space is complex and nonlinear. This requires large data sets to train and utilize the large, complex, nonlinear network architectures required. By contrast, the distribution of 3D facial shapes is well approximated by multivariate Gaussian distributions and amenable to analysis with geometric morphometrics.

Given the differences between 2D- and 3D-based image analysis and increasing affordability of 3D cameras, it is important to explore and validate the potential of 3D imaging for syndrome classification. We show that deep phenotype analysis based on quantitative 3D facial imaging has great potential to facilitate syndrome diagnosis. Furthermore, the accuracy reported here can be improved by integrating other phenotypic data (e.g., a diagnosis of achondroplasia would be incompatible with normal height). For facial and other phenomic data to become clinically useful in the clinic, particularly to assist diagnoses by remote access, it will be necessary to create large, standardized and well-curated data sets of disease characteristics (human phenotype ontologies) and to develop new analytic methods to mine them. To facilitate such efforts, our 3D facial image library and accompanying metadata are consented for data-sharing and are available by application to FaceBase (www.facebase.org).

## Supplementary information

Supplemental Data

Supplementary Movies

Supplementary File S1

Supplementary File S2

Supplementary Video 1

Supplementary Video 2

Supplementary Video 3

Supplementary Video 4

Supplementary Video 5

Supplementary Video 6

Supplementary Video 7

Supplementary Video 8

## Data Availability

Facial images and metadata are available through FaceBase (https://www.facebase.org/chaise/record/#1/isa:data set/accession=FB00000861).

## Code Availability

All R and Python code used in the analysis is available upon request.
