# Supplementary material for: Automated syndrome diagnosis by three-dimensional facial imaging
Source: Genet Med. 2020 Jun 1;22(10):1682–93. doi: 10.1038/s41436-020-0845-y (PMC7521994; doi:10.1038/s41436-020-0845-y)
Supplement: Supplementary file 1 — Supplemental Data [file 41436_2020_845_MOESM1_ESM.pdf]

Supplemental Data

1) ***Figures***

Figure S1: Figure S1: 3D image of the same subject as obtained by a 3dMD photogrammetry system and an iPhone X. A shows a 3dMD mesh and B shows the rendered 3D surface for the same subject obtained from an iPhone X 3D image. This subject has a beard which is much less apparent on the iPhone X image.

Figure S2: 3D landmark set used in the analysis.

Figure S3: Results for the unsymmetrized, symmetrized and combined symmetric and asymmetric components using parametric (CVA) classification. Here, the asymmetric component is the full set of deviations from symmetry re-centered on the sample mean.

Figure S4: Comparison of classification accuracy by machine-learning method.

Figure S5: Comparison of HDRDA validation by LOOCV and 20-fold CV.

Figure S6: Three-dimensional morphs showing the effect for each syndrome compared to the mean shape for unaffecteds. The shape effects shown are 2x the average magnitude to accentuate the anatomical distribution of the differences. The heatmaps show the regions of greatest difference. Blue means that the syndromic mesh is smaller (the surface is behind) and red means that the syndromic mesh is larger (the surface projects beyond).

Figure S7: Syndrome classification by Canonical Variates Analysis (CVA). (A) Classification accuracy by syndrome. For each syndrome, the graph shows the

proportion of syndromes for which the correct diagnosis is the top choice, in the top three, and in the top 10. B) Rankings of correct classifications using HDRDA. A) Counts and proportions for how often the true diagnosis is among the 10 highest ranked diagnoses for the full classification sample that included both syndromic subjects and unrelated, unaffected subjects, and partitioning the true diagnosis ranks as syndromic and unrelated, unaffected subjects.

Figure S8: Matrix of classification probabilities for HDRDA. The results are shown as a heatmap ranging from white (0) to red (1.0). The values that correspond to this plot are provided in File S3. These results show that the highest average classification probability is to the correct syndrome in every case, while the most common misclassifications are to unaffected.

Figure S9: Simulation results for classification tasks with different numbers of syndromes. In each iteration, a group of syndromes (n = 2 to 50) was selected at random from the 65 syndromes included in the main classification analysis. These samples were then subjected to parametric (CVA) classification with cross-validation. The y axis shows the proportion of subjects classified to the correct syndrome and the x axis is the number of syndromes included in the classification task for that iteration.

2) Tables

Table S1: List of patient meetings attended.

Table S2: Race and ethnicity composition of study with comparison to US Census

Table S3: Shape variance attributable to age, sex and race by group

Table S4: Shape variation attributable to syndrome

Table S5: Unweighted average sensitivities across syndromes

Table S6: Analysis of incorrect classification. For each syndrome, this table shows the most common classification errors made by the parametric and HDRDA methods when the correct syndrome was not the top ranked classification. The last column of the table lists the syndrome that is most phenotypically similar (in terms of facial shape) to the correct syndrome.

Table S7: Confusion matrix summaries for HDRDA classification both when including and excluding unrelated unaffected as a class.

3) Files

File S1: Full list of syndromes included in the database

File S2: Analysis of sampling issues with leave-one-out versus k-fold validation.

File S3: Mis-classification plots for all syndromes

File S4-11: Movies of syndromic effects ordered along among-syndrome PCs.

al

Figure S1: 3D image of the same subject as obtained by a 3dMD photogrammetry system and an iPhone X. A shows a 3dMD mesh while B shows the rendered 3D surface for the same subject as obtained from an iPhone X 3D image. This subject has a beard which is much less apparent on the iPhone X image.

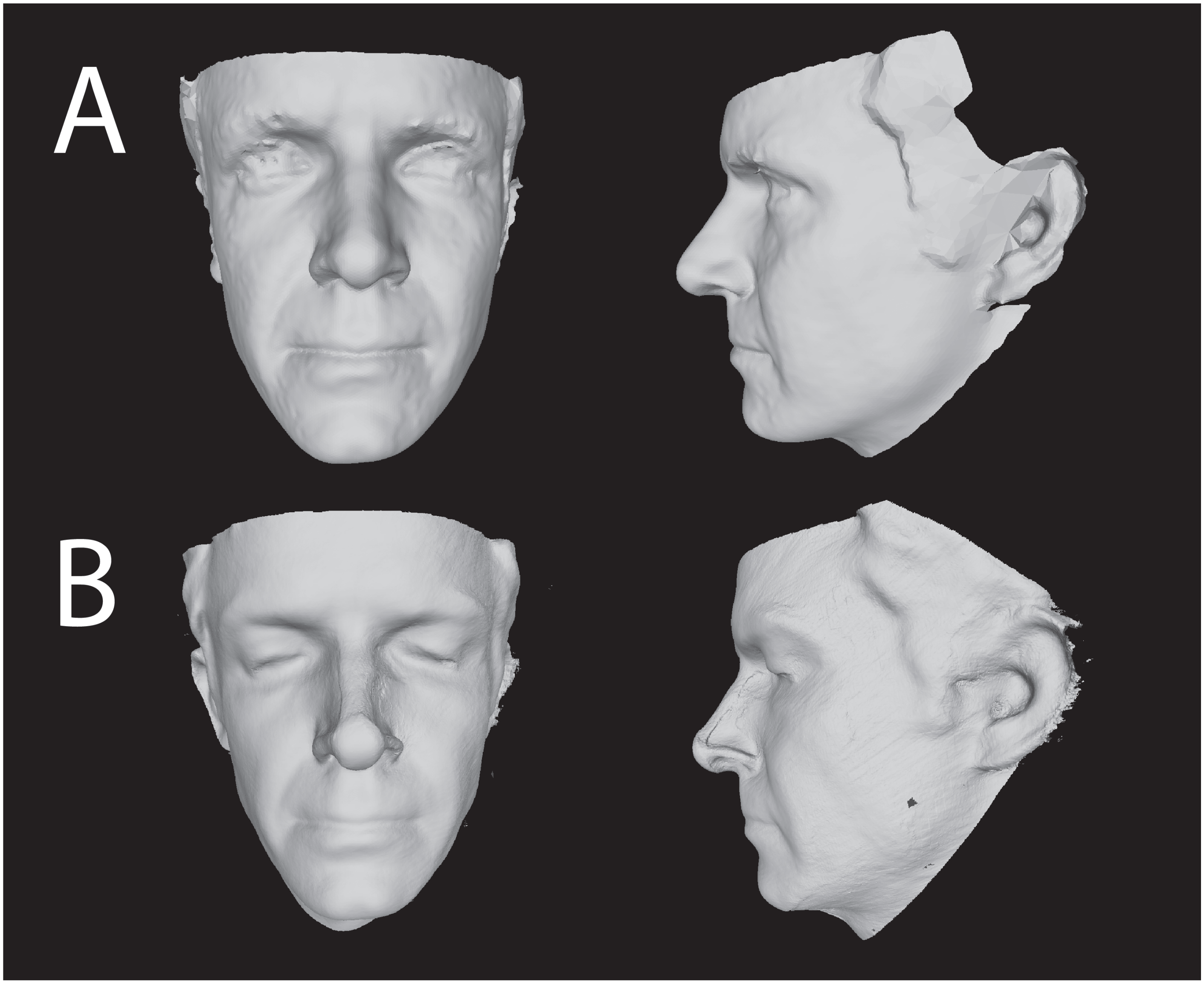

Figure S2: 3D landmark set used in analysis.

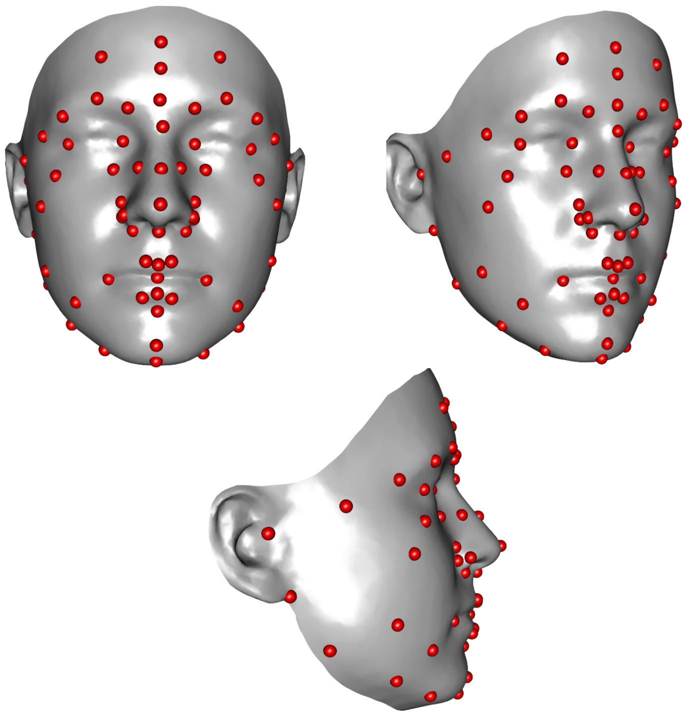

Figure S3: Results for the unsymmetrized, symmetrized and combined symmetric and asymmetric components using parametric (CVA) classification. Here, the asymmetric component is the full set of deviations from symmetry re-centered on the sample mean.

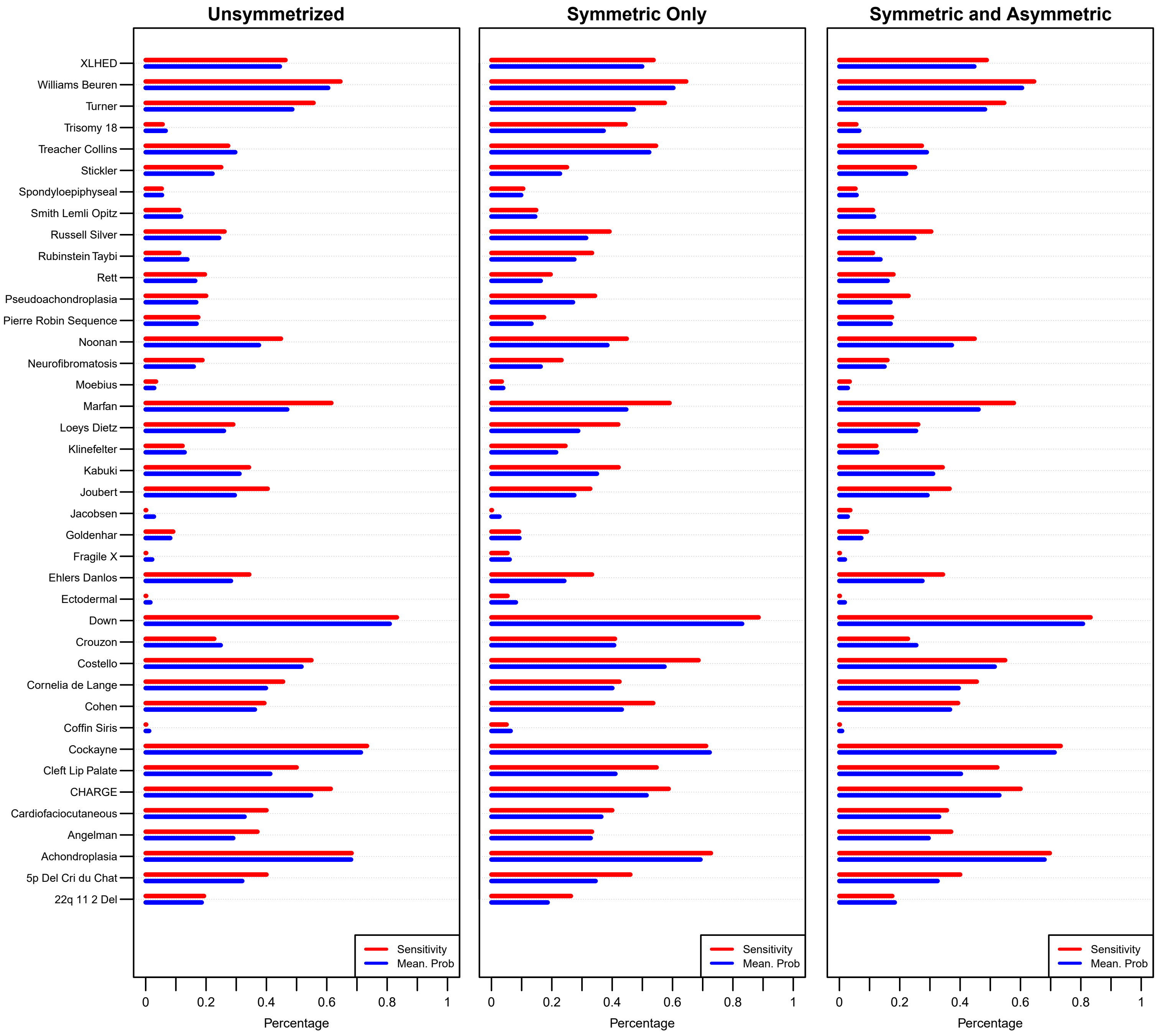

Figure S4: Comparison of sensitivies obtained via different machine-learning methods

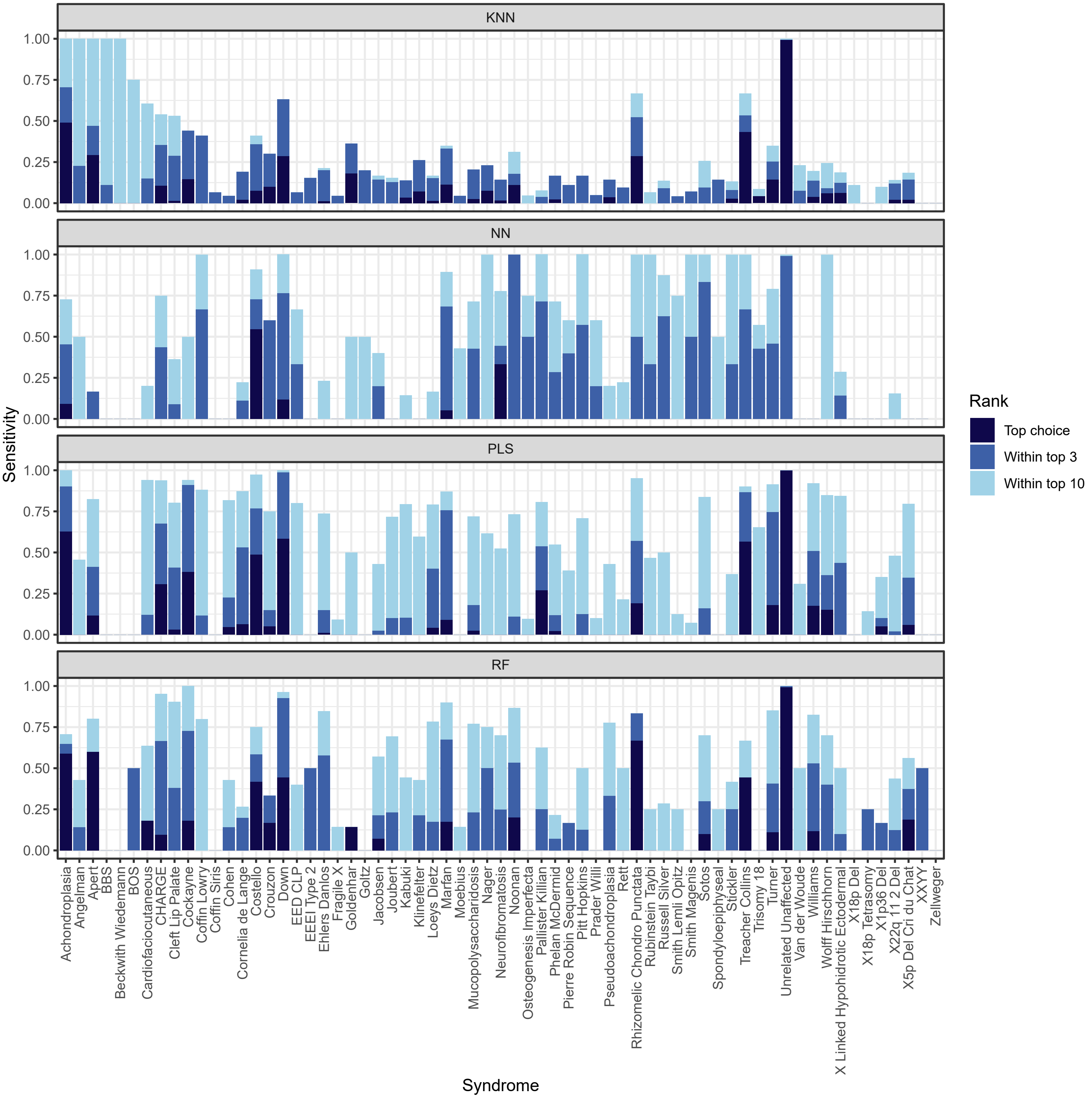

Figure S5: HDRDA Validation by LOOCV and 20-fold CV

A) Leave one out versus 20 fold cross−validation by sample size

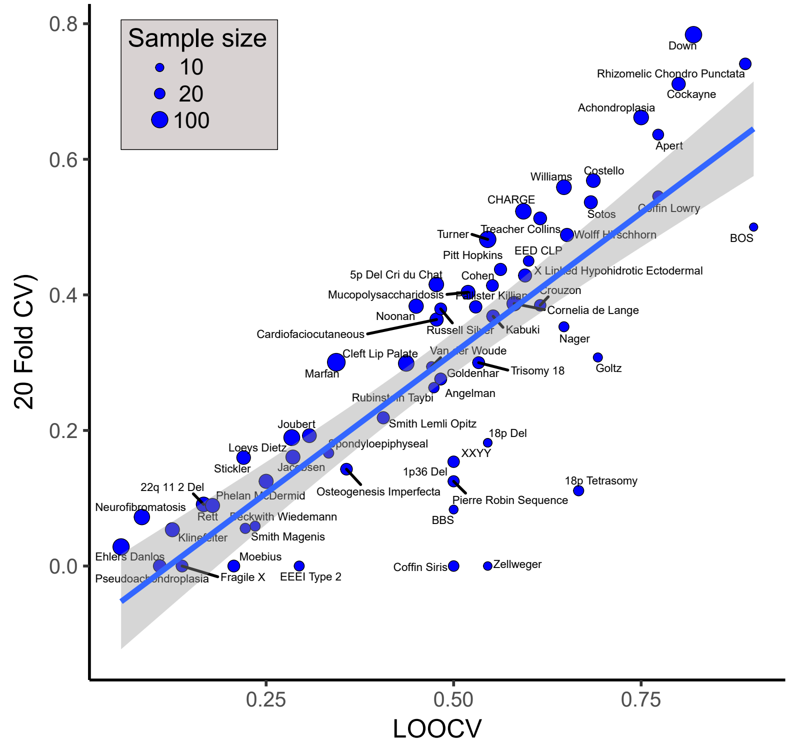

B) Leave one out versus 20 fold cross−validation by Severity

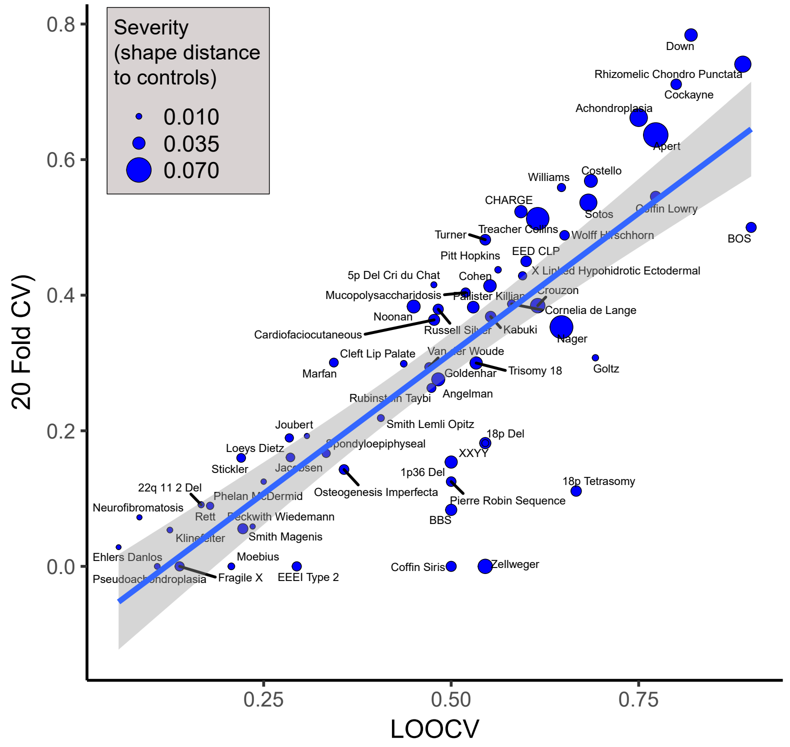

Figure S6: Average shape effects by syndrome

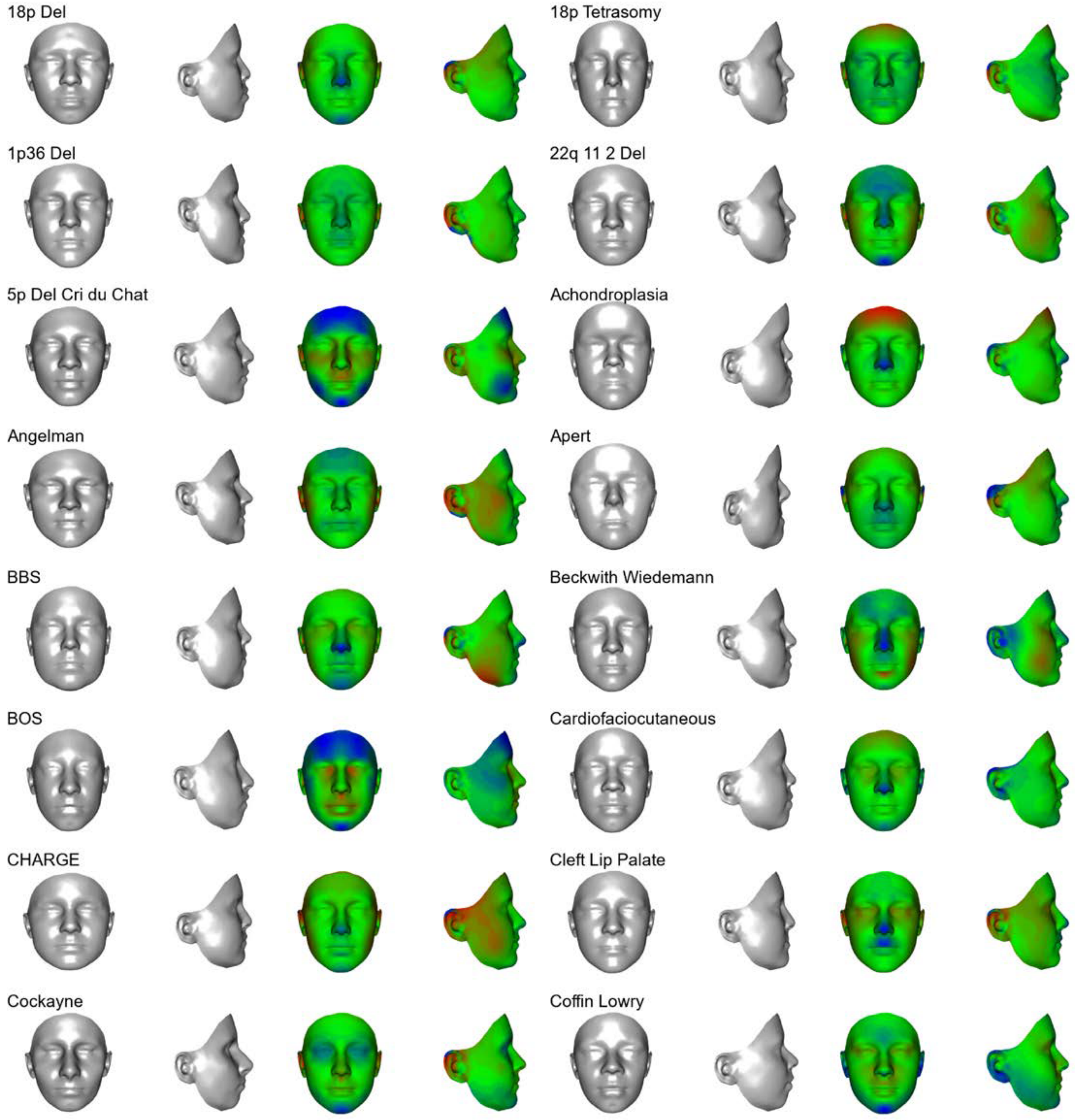

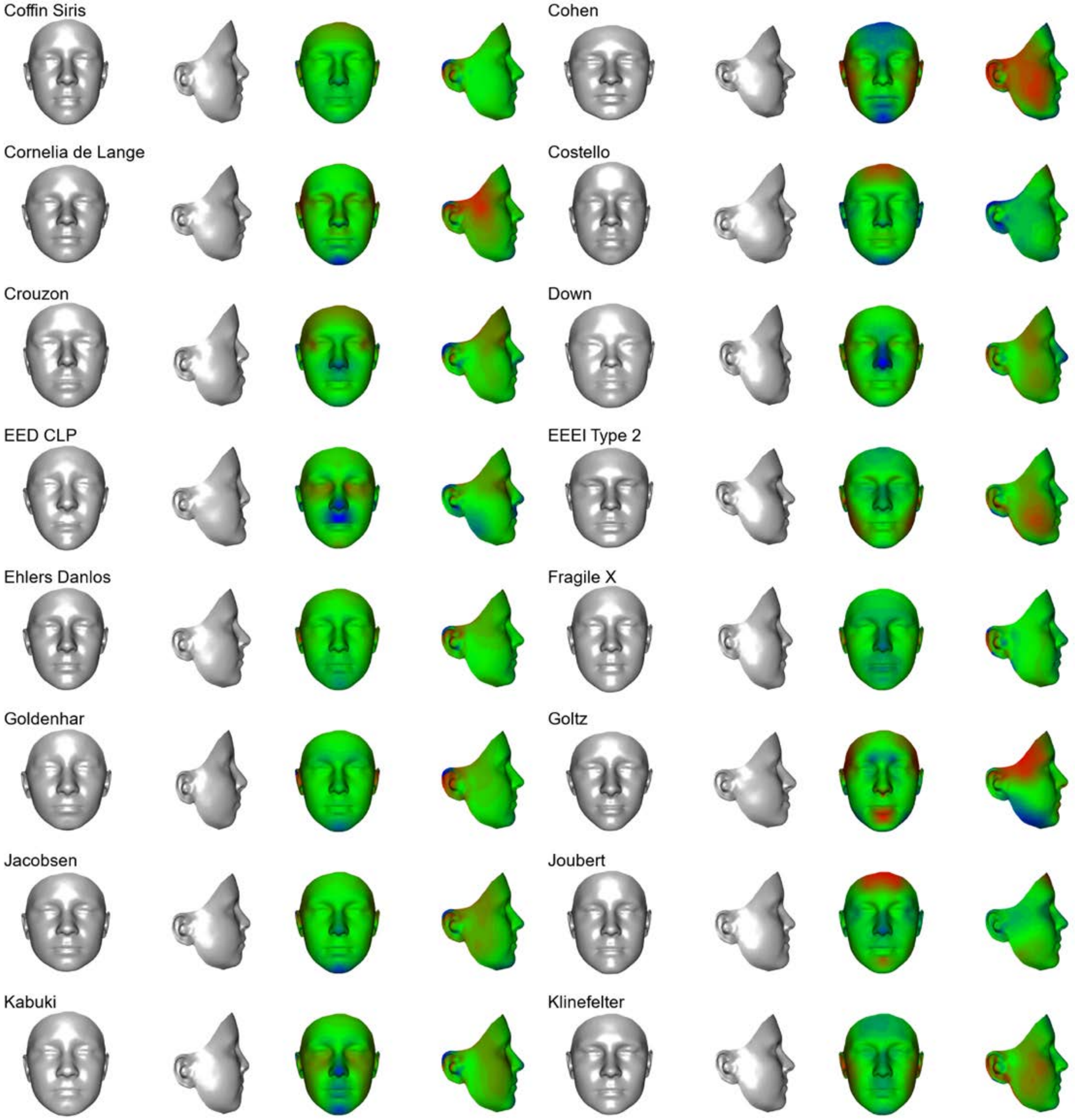

Coffin Siris
Cohen
Cornelia de Lange
Costello
Crouzon
Down
EED CLP
EEEI Type 2
Ehlers Danlos
Fragile X
Goldenhar
Goltz
Jacobsen
Joubert
Kabuki
Klinefelter

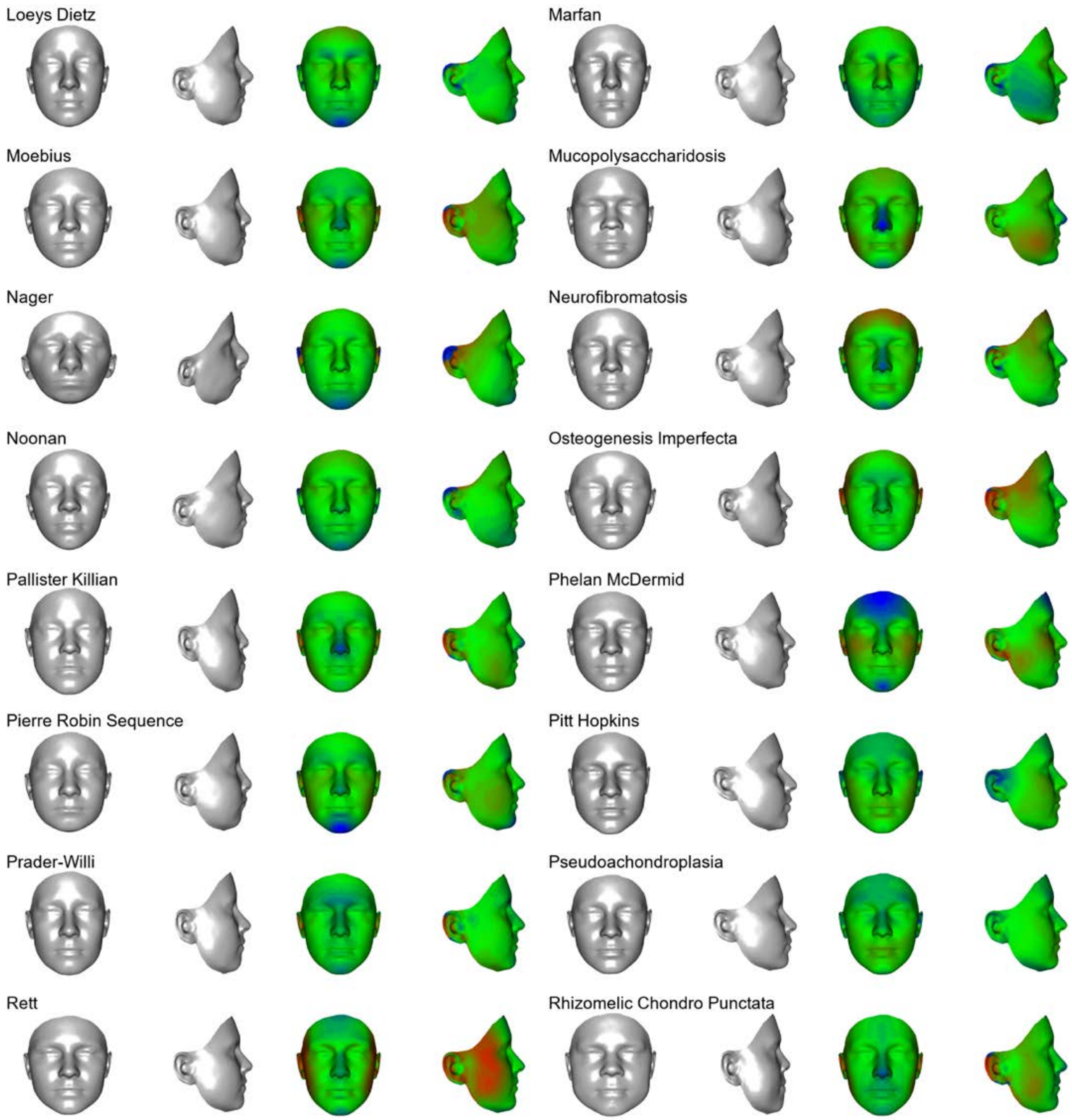
Loeys Dietz
Marfan
Moebius
Mucopolysaccharidosis
Nager
Neurofibromatosis
Noonan
Osteogenesis Imperfecta
Pallister Killian
Phelan McDermid
Pierre Robin Sequence
Pitt Hopkins
Prader-Willi
Pseudoachondroplasia
Rett
Rhizomelic Chondro Punctata

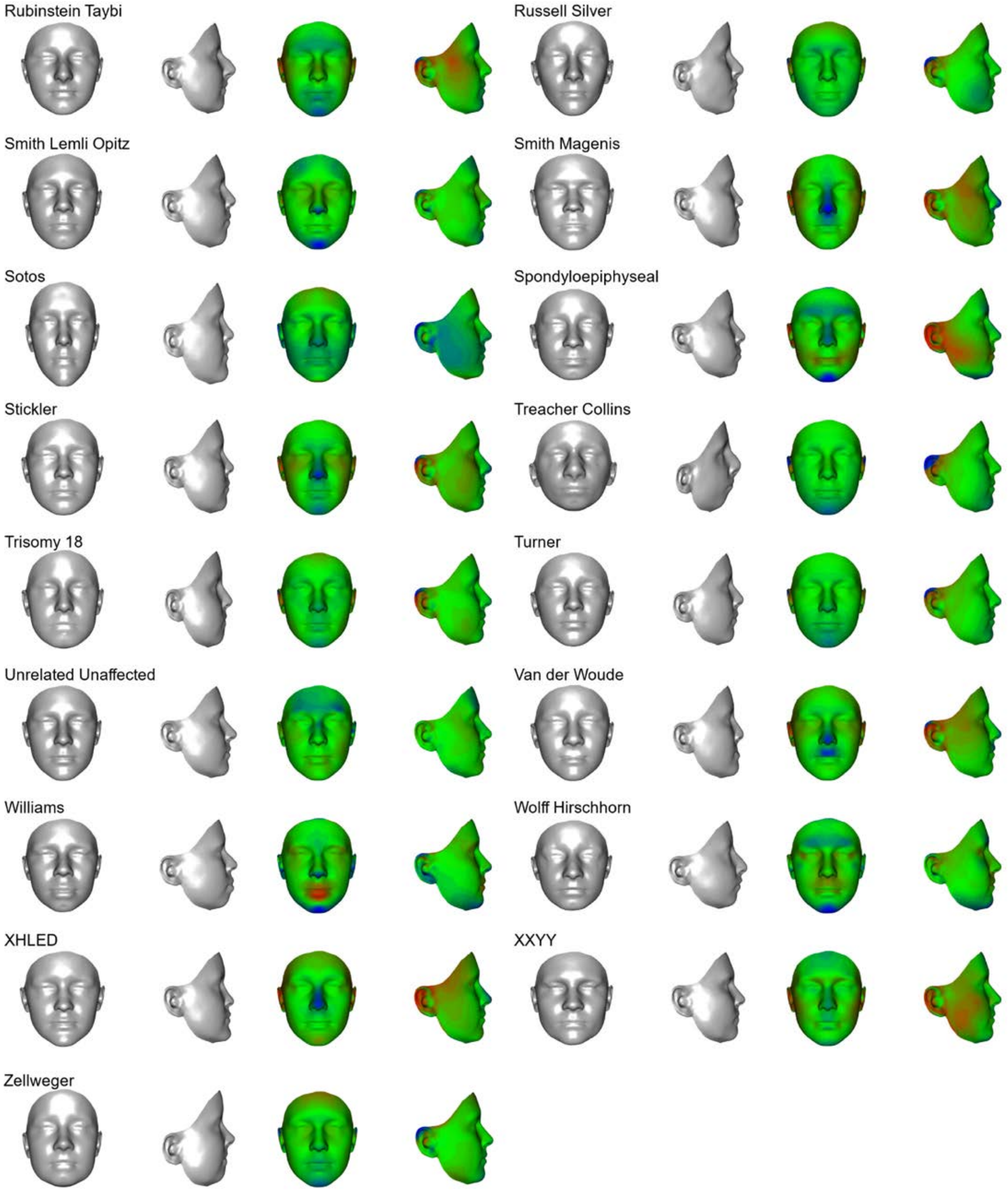

Rubinstein Taybi
Russell Silver
Smith Lemli Opitz
Smith Magenis
Sotos
Spondyloepiphyseal
Stickler
Treacher Collins
Trisomy 18
Turner
Unrelated Unaffected
Van der Woude
Williams
Wolff Hirschhorn
XHLED
XXYY
Zellweger

Figure S7: Syndrome Classification by Canonical Variates Analysis (CVA). (A) Classification accuracy by syndrome. For each syndrome, the graph shows the proportion of syndromes for which the correct diagnosis is the top choice, in the top three, and in the top 10. B) Rankings of Correct Classifications using HDRDA. A) Counts and proportions for how often the true diagoosis is among the 10 highest ranked diagnoses for the full classification sample that included both syndromics subjects and unrelated, unaffected subjects, and partitioning the true diagnosis ranks as syndromic and unrelated, unaffected subjects.

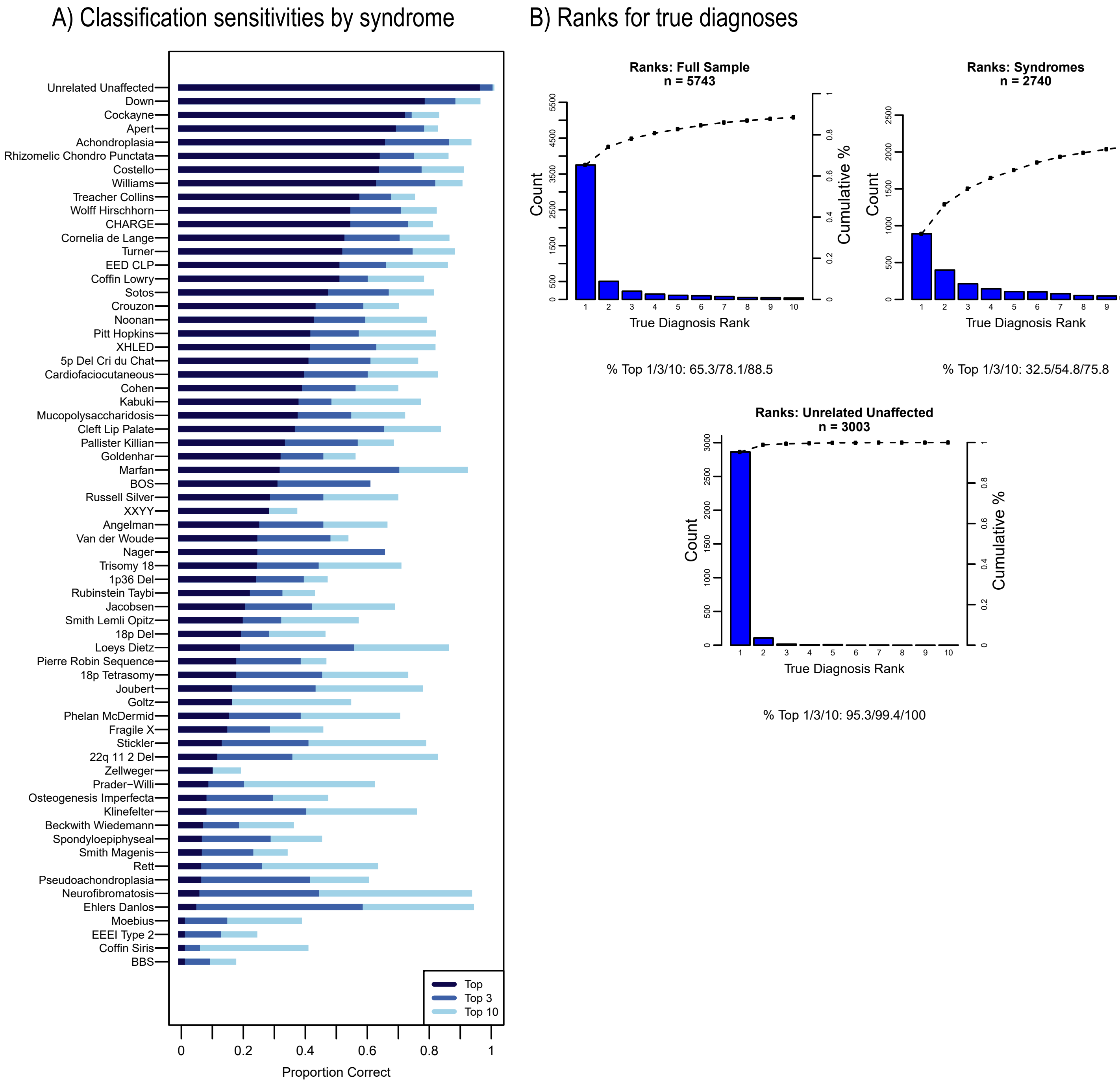

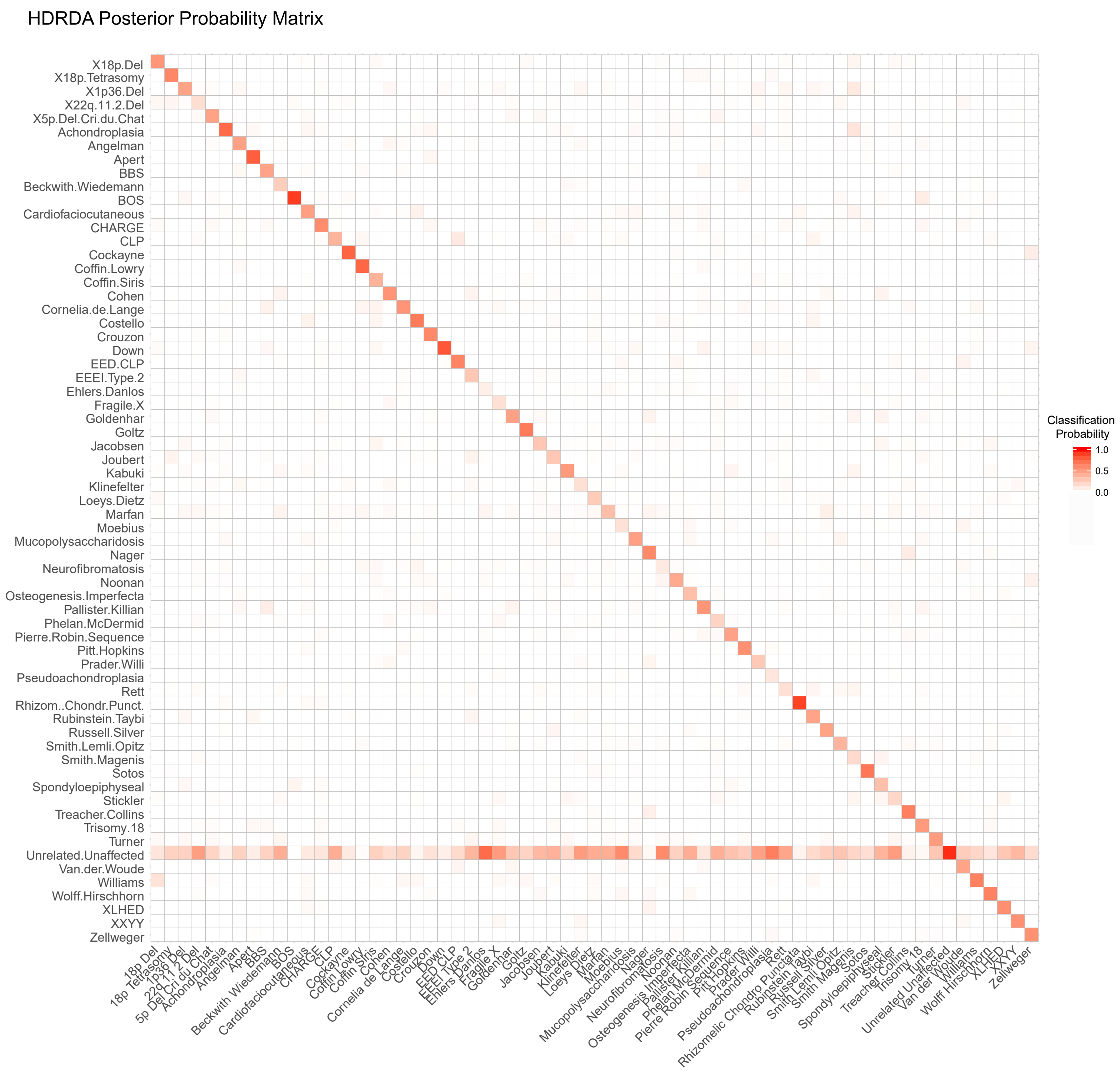

HDRDA Posterior Probability Matrix
X18p.Del
X18p.Tetrasomy
X1p36.Del
X22q.11.2.Del
X5p.Del.Cri.du.Chat
Achondroplasia
Angelman
Apert
BBS
Beckwith.Wiedemann
BOS
Cardiofaciocutaneous
CHARGE
CLP
Cockayne
Coffin.Lowry
Coffin.Siris
Cohen
Cornelia.de.Lange
Costello
Crouzon
Down
EED.CLP
EEEI.Type.2
Ehlers.Danlos
Fragile.X
Goldenhar
Goltz
Jacobsen
Joubert
Kabuki
Klinefelter
Loeys.Dietz
Marfan
Moebius
Mucopolysaccharidosis
Nager
Neurofibromatosis
Noonan
Osteogenesis.Imperfecta
Pallister.Killian
Phelan.McDermid
Pierre.Robin.Sequence
Pitt.Hopkins
Prader.Willi
Pseudoachondroplasia
Rett
Rhizom..Chondr.Punct.
Rubinstein.Taybi
Russell.Silver
Smith.Lemli.Opitz
Smith.Magenis
Sotos
Spondyloepiphyseal
Stickler
Treacher.Collins
Trisomy.18
Turner
Unrelated.Unaffected
Van.der.Woude
Williams
Wolff.Hirschhorn
XLHED
XXYY
Zellweger
18p Del
18p Tetrasomy
1p36 Del
22q 11 2 Del
5p Del Cri du Chat
Achondroplasia
Angelman
Apert
BBS
Beckwith Wiedemann
BOS
Cardiofaciocutaneous
CHARGE
CLP
Cockayne
Coffin Lowry
Coffin Siris
Cohen
Cornelia de Lange
Costello
Crouzon
Down
EED CLP
EEEI Type 2
Ehlers Danlos
Fragile X
Goldenhar
Goltz
Jacobsen
Joubert
Kabuki
Klinefelter
Loeys Dietz
Marfan
Moebius
Mucopolysaccharidosis
Nager
Neurofibromatosis
Noonan
Osteogenesis Imperfecta
Pallister Killian
Phelan McDermid
Pierre Robin Sequence
Pitt Hopkins
Prader Willi
Pseudoachondroplasia
Rett
Rhizomelic Chondro Punctata
Rubinstein Taybi
Russell Silver
Smith Lemli Opitz
Smith Magenis
Sotos
Spondyloepiphyseal
Stickler
Treacher Collins
Trisomy 18
Turner
Unrelated Unaffected
Van der Woude
Williams
Wolff Hirschhorn
XLHED
XXYY
Zellweger
Classification Probability
1.0
0.5
0.0

Figure S8

Figure S9: Relationship between the number of syndromes and top choice accuracy

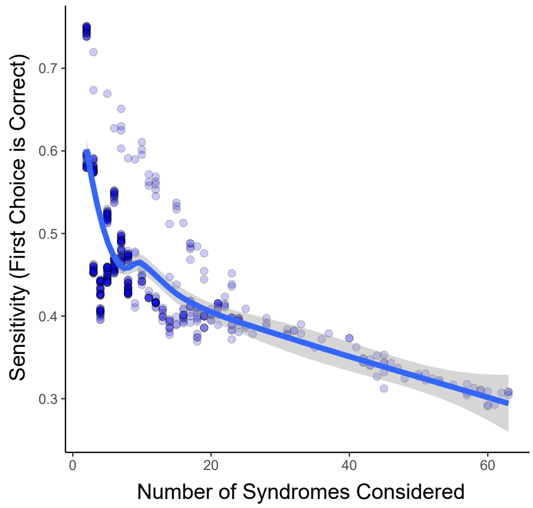

Table S1: Patient meetings attended

Conference	Syndromes	Years Attended
11q Research & Resource Group	Jacobsen Syndrome	2014, 2018
PWS Kids	Prader-Willi Syndrome	2014
CdLS Foundation	Cornelia de Lange Syndrome	2014, 2016, 2018
Stickler Involved People	Stickler Syndrome	2014, 2015, 2016, 2018
4p- Support Group	Wolf-Hirschhorn Syndrome	2014, 2017,2018
National Foundation for Ectodermal Dysplasias	Hypohidrotic Ectodermal Dysplasia<br>X-Linked Hypohidrotic Ectodermal Dysplasia<br>Ectro-dactyly Ectodermal Dysplasia - Cleft Lip/Palate Syndrome<br>Goltz Syndrome	2014, 2015
Kabuki Syndrome Network	Kabuki Syndrome	2014, 2015
Cohen Syndrome Association	Cohen Syndrome	2014, 2016
Little People of America	Achondroplasia<br>Pseudoachondroplasia	2014, 2015, [illegible]
National Downs Syndrome Congress	Trisomy 21	2014, 2015
Turner Syndrome conference	Turner Syndrome	2014
Angelman Syndrome Foundation	Angelman Syndrome	2015, 2017
The Elhers-Danlos Society	Ehlers-Danlos Syndrome	2015
Share & Care Cockyne Syndrome Network	Cockayne Syndrome	2015, 2016, 2017
Chromosome 18 Registry & Research Society	18p-<br>Tetrasomy 18p	2015, 2018
CFC International	Cardio-Facio-Cutaneous Syndrome<br>Costello Syndrome	2015, 2017
The CHARGE Syndrome Foundation	CHARGE Syndrome	2015, 2017
Association for X and Y Chromosome Variations	XXY Syndrome<br>XYY Syndrome<br>XXYY Syndrome<br>XXX Syndrome	2015, 2017
DUP15q Alliance	Dup15q Syndrome	2015
Turner Syndrome Society of the United States	Turner Syndrome	2015
Children's Craniofacial Association	Apert Syndrome<br>Crane-Heise Syndrome<br>Crouzon Syndrome<br>Moebius Syndrome<br>Oropalatal Digital Syndrome<br>Pheiffer Syndrome<br>Sathre-Chotzen Syndrome<br>Treacher Collins Syndrome	2015, 2017, 2018
National Fragile X Foundation	Fragile X Syndrome	2016
Parents and Researchers Interested in Smith-Magenis Syndrome	Smith-Magenis Syndrome	2016
The Marfan Foundation	Marfan Syndrome	2016

	Ehlers-Danlos Syndrome	
Support Organization for Trisomy 18, 13 and Related Disorders	Trisomy 18 Syndrome<br>Mosaic Trisomy 18 Syndrome<br>4p- Deletion Syndrome<br>Trisomy 13 Mosaic Syndrome<br>Trisomy 13 Syndrome	2016, 2017, 2018, 2019
William Syndrome Assocation	William Syndrome	2016
Noonan Syndrome Foundation	Noonan Syndrome	2016
Loeys-Dietz Syndrome Foundation	Loeys-Dietz Syndrome	2016, 2018
5p- Society	Cri du Chat Syndrome	2017, 2018
Costello Syndrome Family Network	Costello Syndrome	2017
Joubert Syndrome and Related Disorders Foundation	Joubert Syndrome	2017
Smith-Lemli-Opitz	RSH Foundation	Smith-Lemli-Opitz Syndrome	2017
Coffin-Lowry Syndrome Foundation	Coffin-Lowry Syndrome	2017, 2018
Rubinstein – Taybi Syndrome Support Group	Rubenstein-Taybi Syndrome	2017
Coffin-Siris Syndrome Foundation	Coffin-Siris Syndrome	2017, 2018
RhizoKids International	Rhizomelic Chondrodysplasia Punctata	2017, 2018
Global Foundation for Peroxisomal Disorders	Peroxisomal Biogenesis Disorder	2017
Foundation for Prader-Willi Research	Prader-Willi Syndrome	2017
The MAGIC Foundation	McCune Albright Syndrome<br>Russell Silver Syndrome	2017, 2018
Foundation for Nager & Miller Syndromes	Nager Syndrome<br>Miller Syndrome	2017
Pitt Hopkins Research Foundation	Pitt Hopkins Syndrome	2018
PKS Kids	Pallister-Killian Syndrome	2018
MPS Society	Mucopolysaccharidosis	2018
Sotos Syndrome Support Association	Sotos Syndrome	2018
Phelan-McDermid Syndrome Foundation	Phelan-McDermid Syndrome	2018
The Bohring-Opitz Syndrome Foundation, Inc.	Bohring-Opitz Syndrome	2018
1p36 Deletion Support & Awareness	1p36 Deletion Syndrome	2018
Bardet Biedl Syndrome Foundation	Bardet Biedl Syndrome	2018
ASXL Conference	Borhing-Opiz Syndrome	2019
	Bainbridge-Ropers Syndrome	
	Sashi-Pena Syndrome	
Bohring-Opitz Family Meeting	Borhing-Opiz Syndrome	2019
DYRK1A Seattle Meet-up June	DYRK1A-Related Syndrome	2019
Bardet Biedl Syndrome Foundation	Bardet Biedl Syndrome	2018
Neurofibromatosis Callifornia	Neurofibromatosis Type 1	2018
Soft Bones-The US Hypophosphatasia Foundation	The US Hypophosphatasia Foundation	2019

	US Census 2010	FB2 syndromic subjects and relatives		
Race (NIH Classification)	Percentage	Count	Percentage including not reported	Percentage excluding not reported
White	76.5%	2684	66.2%	83.1%
Black/African American	13.4%	54	1.3%	1.7%
American Indian/Alaska Native	1.3%	12	0.3%	0.4%
Asian	5.9%	102	2.5%	3.2%
Native Hawaiian/Pacific Islander	0.2%	1	0.0%	0.0%
More than one race	2.7%	375	9.3%	11.6%
Unknown or not reported	NA	826	20.4%	
Total		4054		
Excluding not reported		3228		

Ethnicity (NIH Classification)	Percentage	Count	Percentage including not reported	Percentage excluding not reported
Hispanic/Latino	18.3	422	10.4%	12.7%
Not Hispanic/Latino	81.7	2906	71.7%	87.3%
unknown/not reported	NA	726	17.9%	
Total		4054		
Excluding not reported		3328		

Table S3: RRPP MANCOVA for facial shape on age, sex and self-reported race

1) Syndromic subjects only

	Df	SS	MS	Rsq	F	Z	*p*
Age	1	1.35	1.35	0.093	368.05	10.65	<0.001
Age2	1	0.58	0.58	0.040	159.76	10.10	<0.001
Age3	1	0.16	0.16	0.011	43.02	8.06	<0.001
Sex	1	0.09	0.09	0.007	25.89	7.32	<0.001
Race	7	0.15	0.02	0.011	6.05	9.25	<0.001
Residuals	3315	12.12	0.00	0.838			
Total	3326	14.46					

2) Unaffected, unrelated subjects only

	Df	SS	MS	Rsq	F	Z	*p*
Age	1	1.28	1.28	0.189	798.01	11.31	<0.001
Age2	1	0.35	0.35	0.051	215.50	10.82	<0.001
Age3	1	0.11	0.11	0.017	71.12	9.01	<0.001
Sex	1	0.13	0.13	0.019	82.25	9.59	<0.001
Race	6	0.11	0.02	0.016	11.22	11.80	<0.001
Residua	2992	4.80	0.00	0.708			
Total	3002	6.78					

*Units are in Procrustes distance

Table S4: Facial shape variance due to syndrome

	Df	SS	MS	Rsq	F	Z	*p*
1) Symmetrized Data including unrelated unaffecteds							
Syndrome	64	2.059	0.032	0.148	15.45	24.30	<0.001
Residuals	5678	11.822	0.002	0.852			
2) Full Procrustes Data including unrelated unaffecteds							
Syndrome	64	2.097	0.033	0.138	14.18	24.81	<0.001
Residuals	5678	13.121	0.002	0.862			
3) Symmetrized Data excluding unrelated unaffecteds							
Syndrome	63	1.669	0.026	0.186	9.73	22.12	<0.001
Residuals	2675	7.276	0.003	0.812			
4) *Full Procrustes Data excluding unrelated unaffecteds*							
Syndrome	63	1.669	0.026	0.186	9.73	22.12	<0.001
Residuals	2676	7.287	0.003	0.814			

*Units are in Procrustes distance

Table S5: Unweighted average sensitivities across syndromes

	Top 1	Top 3	Top 10
1) *CVA*			
Full classification sample (syndromic subjects and unrelated, unaffected subjects)	30.0%	48.3%	67.3%
Syndrome-only classification sample	34.0%	52.0%	68.0%
2) *HDRDA*			
Full classification sample (syndromic subjects and unrelated, unaffected subjects)	48.8%	69.3%	87.2%
Syndrome-only classification sample	56.9%	73.9%	88.5%

Syndrome	N	Parametric Classification				HDRDA Classification				Most Similar Syndrome (Minimum Procrustes Distance)
		Most Frequent classification error	%	Next miss	%	Most Frequent Misdiagnosis	%	Next miss	%	
18p Del	11	Williams	27%	22q 11 2 Del	18%	Unrelated Unaffected	18%	Williams	18%	Stickler
18p Tetrasomy	18	Unrelated Unaffected	56%	Joubert	11%	Unrelated Unaffected	28%	Joubert	6%	Coffin Siris
1p36 Del	26	Unrelated Unaffected	31%	Pitt Hopkins	8%	Unrelated Unaffected	27%	BOS	4%	Fragile X
22q 11 2 Del	66	Unrelated Unaffected	58%	5p Del Cri du Chat	3%	Unrelated Unaffected	55%	Joubert	3%	Klinefelter
5p Del Cri du Chat	65	Unrelated Unaffected	26%	Angelman	5%	Unrelated Unaffected	23%	CHARGE	3%	Phelan McDermid
Achondroplasia	68	Unrelated Unaffected	9%	Cardiofaciocutaneous	4%	Unrelated Unaffected	7%	Mucopolysaccharido	3%	Crouzon
Angelman	29	Unrelated Unaffected	31%	Prader-Willi	7%	Unrelated Unaffected	24%	BBS	3%	Rett
Apert	22	Achondroplasia	9%	Rubinstein Taybi	9%	Unrelated Unaffected	9%	Achondroplasia	5%	Crouzon
BBS	12	Unrelated Unaffected	25%	CHARGE	17%	Unrelated Unaffected	25%	CHARGE	8%	Stickler
Beckwith Wiedemann	17	Unrelated Unaffected	65%	Cohen	6%	Unrelated Unaffected	47%	Cohen	6%	Unrelated Unaffected
BOS	10	Unrelated Unaffected	40%	Pallister Killian	10%	Spondyloepiphyseal	10%	Achondroplasia	0%	5p Del Cri du Chat
Cardiofaciocutaneous	44	Unrelated Unaffected	14%	Costello	11%	Unrelated Unaffected	11%	Costello	7%	Costello
CHARGE	86	Unrelated Unaffected	16%	Down	2%	Unrelated Unaffected	12%	Achondroplasia	2%	Jacobsen
Cleft Lip Palate	87	Unrelated Unaffected	38%	Jacobsen	3%	Unrelated Unaffected	45%	Van der Woude	3%	Van der Woude
Cockayne	45	Unrelated Unaffected	11%	Klinefelter	4%	Unrelated Unaffected	9%	Angelman	2%	5p Del Cri du Chat
Coffin Lowry	22	Unrelated Unaffected	9%	Williams	9%	BBS	5%	Cleft Lip Palate	5%	Williams
Coffin Siris	20	Unrelated Unaffected	40%	Williams	10%	Unrelated Unaffected	25%	Cornelia de Lange	5%	Marfan
Cohen	29	Unrelated Unaffected	17%	1p36 Del	7%	Unrelated Unaffected	17%	Angelman	3%	Rett
Cornelia de Lange	62	Unrelated Unaffected	23%	Williams	6%	Unrelated Unaffected	23%	Pitt Hopkins	5%	Jacobsen
Costello	51	Cardiofaciocutaneous	12%	Unrelated Unaffected	6%	Cardiofaciocutaneous	6%	Neurofibromatosis	6%	Cardiofaciocutaneous
Crouzon	26	Unrelated Unaffected	19%	Apert	12%	Unrelated Unaffected	19%	Achondroplasia	4%	18p Del
Down	111	Unrelated Unaffected	8%	1p36 Del	2%	Unrelated Unaffected	12%	1p36 Del	2%	Smith Magenis
EED CLP	20	Unrelated Unaffected	25%	Cleft Lip Palate	15%	Unrelated Unaffected	20%	Cleft Lip Palate	15%	Cleft Lip Palate
EEEI Type 2	17	Unrelated Unaffected	65%	Rett	12%	Unrelated Unaffected	47%	Cohen	6%	Rett
Ehlers Danlos	106	Unrelated Unaffected	80%	Marfan	5%	Unrelated Unaffected	78%	Marfan	5%	Neurofibromatosis
Fragile X	29	Unrelated Unaffected	62%	1p36 Del	3%	Unrelated Unaffected	59%	Cornelia de Lange	3%	Prader-Willi
Goldenhar	29	Unrelated Unaffected	34%	1p36 Del	3%	Unrelated Unaffected	31%	Pallister Killian	7%	Turner
Goltz	13	Unrelated Unaffected	38%	Noonan	15%	Unrelated Unaffected	23%	Williams	8%	Unrelated Unaffected

Jacobsen	56	Unrelated Unaffected	41%	5p Del Cri du Chat	5%	Unrelated Unaffected	43%	X5p Del Cri du Chat	5%	Cornelia de Lange
Joubert	52	Unrelated Unaffected	46%	Fragile X	4%	Unrelated Unaffected	42%	Russell Silver	6%	Neurofibromatosis
Kabuki	38	Unrelated Unaffected	29%	CHARGE	5%	Unrelated Unaffected	24%	Stickler	5%	Stickler
Klinefelter	56	Unrelated Unaffected	50%	Angelman	7%	Unrelated Unaffected	59%	1p36 Del	5%	22q 11 2 Del
Loeys Dietz	95	Unrelated Unaffected	45%	Marfan	3%	Unrelated Unaffected	44%	Marfan	3%	Ehlers Danlos
Marfan	163	Unrelated Unaffected	47%	Noonan	2%	Unrelated Unaffected	45%	Ehlers Danlos	3%	Loeys Dietz
Moebius	29	Unrelated Unaffected	79%	Cardiofaciocutaneous	3%	Unrelated Unaffected	62%	Cardiofaciocutaneou	3%	22q 11 2 Del
Mucopolysaccharidosis	52	Unrelated Unaffected	29%	Achondroplasia	6%	Unrelated Unaffected	21%	Down	4%	Jacobsen
Nager	17	Treacher Collins	41%	Goldenhar	6%	Goldenhar	6%	Marfan	6%	Treacher Collins
Neurofibromatosis	83	Unrelated Unaffected	66%	Ehlers Danlos	4%	Unrelated Unaffected	69%	Costello	4%	Ehlers Danlos
Noonan	60	Unrelated Unaffected	27%	Cardiofaciocutaneous	5%	Unrelated Unaffected	25%	EED CLP	5%	Cardiofaciocutaneous
Osteogenesis Imperfecta	28	Unrelated Unaffected	54%	18p Tetrasomy	4%	Unrelated Unaffected	43%	Ehlers Danlos	4%	Moebius
Pallister Killian	34	Unrelated Unaffected	12%	Cornelia de Lange	9%	Unrelated Unaffected	15%	Down	6%	Fragile X
Phelan McDermid	56	Unrelated Unaffected	48%	5p Del Cri du Chat	5%	Unrelated Unaffected	39%	5p Del Cri du Chat	5%	22q 11 2 Del
Pierre Robin Sequence	24	Unrelated Unaffected	50%	CHARGE	8%	Unrelated Unaffected	33%	Kabuki	8%	Moebius
Pitt Hopkins	32	Unrelated Unaffected	25%	22q 11 2 Del	3%	Unrelated Unaffected	28%	Rett	6%	Pseudoachondroplasia
Prader-Willi	26	Unrelated Unaffected	58%	1p36 Del	4%	Unrelated Unaffected	54%	Achondroplasia	4%	Fragile X
Pseudoachondroplasia	37	Unrelated Unaffected	68%	Mucopolysaccharidosis	5%	Unrelated Unaffected	70%	Cornelia de Lange	3%	Pitt Hopkins
Rett	56	Unrelated Unaffected	46%	Klinefelter	7%	Unrelated Unaffected	52%	1p36 Del	5%	XXYY
Rhizomelic Chondro Punct	27	Unrelated Unaffected	11%	Cardiofaciocutaneous	4%	Unrelated Unaffected	7%	Cardiofaciocutaneou	4%	Smith Magenis
Rubinstein Taybi	19	Unrelated Unaffected	42%	Cleft Lip Palate	5%	Unrelated Unaffected	16%	Cleft Lip Palate	5%	Moebius
Russell Silver	29	Unrelated Unaffected	31%	Marfan	7%	Unrelated Unaffected	31%	Marfan	7%	Turner
Smith Lemli Opitz	32	Unrelated Unaffected	41%	Mucopolysaccharidosis	6%	Unrelated Unaffected	38%	Mucopolysaccharidc	6%	Ehlers Danlos
Smith Magenis	18	Unrelated Unaffected	22%	Achondroplasia	17%	Unrelated Unaffected	22%	Achondroplasia	11%	Down
Sotos	41	Unrelated Unaffected	22%	Marfan	7%	Unrelated Unaffected	17%	Achondroplasia	2%	18p Tetrasomy
Spondyloepiphyseal	18	Unrelated Unaffected	61%	Stickler	11%	Unrelated Unaffected	39%	Cohen	6%	Rett
Stickler	50	Unrelated Unaffected	60%	Marfan	4%	Unrelated Unaffected	56%	Turner	4%	Moebius
Treacher Collins	39	Nager	13%	Unrelated Unaffected	13%	Nager	10%	Unrelated Unaffecte	8%	Nager
Trisomy 18	30	Unrelated Unaffected	13%	BOS	7%	BOS	10%	Angelman	3%	Coffin Siris
Turner	110	Unrelated Unaffected	30%	Down	2%	Unrelated Unaffected	29%	Smith Lemli Opitz	2%	Pierre Robin Sequence
Unrelated Unaffected	3003	Loeys Dietz	0.4%	Turner	0.4%					Ehlers Danlos

Van der Woude	17	Unrelated Unaffected	29%	Cleft Lip Palate	24%	Unrelated Unaffected	29%	EED CLP	6%	Cleft Lip Palate
Williams	68	Unrelated Unaffected	22%	Marfan	4%	Unrelated Unaffected	22%	Cornelia de Lange	4%	Neurofibromatosis
Wolff Hirschhorn	43	Unrelated Unaffected	12%	Trisomy 18	7%	Unrelated Unaffected	12%	Jacobsen	5%	Cornelia de Lange
XHLED	42	Unrelated Unaffected	36%	Down	5%	Unrelated Unaffected	29%	Stickler	5%	Rett
XXYY	11	Unrelated Unaffected	64%	Klinefelter	9%	Unrelated Unaffected	36%	Klinefelter	9%	Klinefelter
Zellweger	11	Unrelated Unaffected	27%	Cleft Lip Palate	9%	Unrelated Unaffected	18%	Cockayne	9%	Kabuki
