# Supplementary material for: Automated syndrome diagnosis by three-dimensional facial imaging
Source: Genet Med. 2020 Jun 1;22(10):1682–93. doi: 10.1038/s41436-020-0845-y (PMC7521994; doi:10.1038/s41436-020-0845-y)
Supplement: Supplementary file 2 — Supplementary Movies [file 41436_2020_845_MOESM2_ESM.pdf]

Sample Size	Syndrome
916	Relative
190	Cleft Lip/Palate
189	Marfan Syndrome
162	Down Syndrome
157	Unaffected Unrelated
123	Turner Syndrome
117	Ehlers Danlos Syndrome
105	CHARGE Syndrome
101	Loeys-Dietz Syndrome
97	Cornelia de Lange Syndrome
96	Neurofibromatosis
93	22q 11.2 Deletion Syndrome
86	Jacobsen Syndrome
83	Achondroplasia
78	5p Deletion Cri du Chat
76	Williams-Beuren Syndrome
74	Klinefelter Syndrome
72	Phelan McDermid Syndrome
69	Noonan Syndrome
69	Wolf-Hirschhorn syndrome
63	Treacher Collins Syndrome
59	Mucopolysaccharidosis
59	Stickler Syndrome
56	Joubert Syndrome
52	Costello Syndrome
52	Rett Syndrome
51	Pallister-Killian Syndrome
51	Sotos Syndrome
48	Cockayne Syndrome
47	Cardiofaciocutaneous Syndrome
47	Kabuki Syndrome
46	X-Linked Hypohidrotic Ectodermal Dysplasia
42	Goldenhar Syndrome
39	Pierre Robin Sequence
38	Pseudoachondroplasia
38	Smith-Lemli-Opitz Syndrome
37	Osteogenesis Imperfecta
36	Apert Syndrome
36	Crouzon Syndrome
36	Trisomy 18
35	Angelman Syndrome
35	Prader-Willi Syndrome
35	Rhizomelic Chondrodysplasia Punctata
34	1p36 Deletion
34	Moebius Syndrome
34	Wiedemann-Steiner Syndrome

33	Cohen Syndrome
33	Fragile X
33	Hypophosphatasia
33	Pitt-Hopkins Syndrome
33	Russell Silver Syndrome
26	Rubinstein-Taybi Syndrome
24	Coffin-Siris Syndrome
24	Ectrodactyly-Ectodermal Dysplasia-Cleft Lip/Palate
23	Coffin-Lowry Syndrome
22	Nager Syndrome
21	Epileptic Encephalopathy Early Infantile Type 2
21	Spondyloepiphyseal Dysplasia
20	18p Tetrasomy
20	Beckwith-Weidemann Syndrome
20	Bohring-Opitz Syndrome
20	DYRK1A-Related Intellectual Disability Syndrome
20	Ectodermal Dysplasia
19	Hemifacial Microsomia
19	Smith-Magenis Syndrome
19	Van der Woude Syndrome
17	Saethre-Chotzen Syndrome
17	XXYY
15	Bardet-Biedl Syndrome
14	Bainbridge-Ropers Syndrome
14	Goltz Syndrome
14	Pfeiffer Syndrome
14	Zellweger Syndrome
13	18p Deletion
13	Rett Syndrome_Other
13	Septo-Optic Dysplasia
12	Branchio-Oto-Renal Syndrome
11	Craniofrontonasal Dysplasia
11	XXX
10	15q Duplication
10	Hypochondroplasia
10	Trisomy 13
10	VACTERL
9	22q11 Duplication
9	Growth Hormone Deficiency
9	Skeletal Dysplasia
8	Diastrophic Dysplasia
8	Gaucher Disease
8	Simpson-Golabi-Behmel Syndrome
8	XYY
7	15q11.2 Deletion
7	Muenke Syndrome
7	Robinow Syndrome

6	Cleidocranial Dysostosis
6	McCune Albright Syndrome
6	Miller Syndrome
6	Opitz GBBB Syndrome
5	15q26.3 Deletion
5	16p11.2 Duplication
5	Frontonasal Dysplasia
5	Parry-Romberg Syndrome
5	PHACE Syndrome
5	Phenylketonuria
5	Primordial Dwarfism
5	Tuberous Sclerosis
5	Xq28 Duplication
4	16p11.2 Deletion
4	1q21.1 Deletion
4	1q21.1 Duplication
4	CLOVES Syndrome
4	Hydrotic Ectodermal Dysplasia
4	KBG syndrome
4	Microcephaly
4	Mowat-Wilson Syndrome
4	Potocki-Lupski Syndrome
4	Rett Syndrome_CDKL5
4	Trisomy 22
4	Waardenburg Syndrome
3	10q21.3 Deletion
3	15q 13.3 Duplication
3	15q Deletion
3	15q11.2 Duplication
3	15q13.3 Deletion
3	18q Deletion
3	21q22 Deletion
3	2q37 Deletion
3	3p.26.3 Deletion
3	3q.29 Deletion
3	Blepharocheilodontic Syndrome
3	Campomelic Dysplasia
3	Dilated Cardiomyopathy and Ataxia Syndrome
3	Ellis-van Creveld Syndrome
3	Epileptic Encephalopathy
3	Guanidinoacetate Methyltransferase Deficiency
3	Hurler Syndrome
3	Hypohidrotic Ectodermal Dysplasia
3	Macrocephaly
3	medium chain acyl-CoA dehydrogenase deficiency
3	Nail-Patella Syndrome
3	Oculocutaneous Albinism

3	Otopalatodigital Syndrome
3	Popliteal Pterygium Syndrome
3	Roberts Syndrome
3	Spondyloepipmetaphyseal Dysplasia
3	Velopharyngeal Insufficiency
3	Xp22.3 Deletion
2	12p11.22q11 Duplication
2	13q21.32 Deletion
2	16q13 Deletion
2	19p13 Deletion
2	19q13 Duplication
2	1q44 Deletion
2	2 Partial Deletion
2	3q25 Duplication
2	3q26 Deletion
2	4q13 Duplication
2	4q31 Deletion
2	7q21.3 Deletion
2	8p Inversion, Duplication, Deletion
2	9p23 Deletion
2	9p24.3 Deletion
2	Alagille syndrome
2	Alport Syndrome
2	Andersen cardiodysrhythmic period paralysis
2	Beals Syndrome
2	Borjeson-Forssman-Lehmann Syndrome
2	Branchiooculofacial syndrome
2	Cerebrocostomandibular Syndrome
2	Chromosomal Abnormality
2	Chrondrodysplasia Punctata
2	Crane-Heise Syndrome
2	Ea1 Dystonia
2	Fabry Disease
2	Femoral-Facial Syndrome
2	GLASS Syndrome
2	hereditary diffuse gastric cancer
2	intellectual disability
2	Intellecutal Developmental Disability Disorder with Dysmorphic Facies and Ptosis
2	Isodicentric 15
2	Kleefstra Syndrome
2	Klippel-Feil Syndrome
2	Kniest dysplasia
2	LADD Syndrome
2	Lissencephaly
2	Mandibulofacial Dysostosis Microcephaly Syndrome
2	Marshall-Smith Syndrome
2	MED12 Syndrome

2	MN1 Mutation
2	NSDHL-related disorder
2	Oculocerebrocutaneous Syndrome
2	Odontoonychodermal Dysplasia
2	Ornithine transcarbamylase deficiency
2	Osteopetrosis
2	Rett Syndrome_FOXG1
2	Spondylocostal Dysplasia
2	Spondylometaphyseal Dysplasia
2	Teebi Hypertelorism Syndrome
2	Townes-Brocks Syndrome
2	Trichorhinophalangeal Syndrome
2	Trichothiodystrophy
2	Trisomy 16
2	Trisomy 8
2	Trisomy 9
2	Xp22.31 Duplication
1	10p11.2q21.3 Inversion
1	10q22.3 Deletion
1	10q24.32-q25.1 Deletion
1	10q25.1 Deletion
1	10q25.2-q26.11 Deletion
1	10q25.3 Duplication
1	10q26 Deletion
1	11;16 Translocation
1	11q13.4-13.5 deletion
1	11q14.1 Duplication
1	11q24.1 or 2 Deletion
1	12p Deletion
1	12q21.23 Deletion; 11p14.2p14.1 Duplication
1	12q24.31 Duplication; 16p13.11-16p12.3 Deletion
1	12q24.32 anomaly
1	13q Deletion
1	13q12.3 - 13.3 Deletion
1	14q11.2 Deletion
1	14q11.2 Deletion/16p11.2 Duplication/17q21.4 Duplication
1	15p Deletion
1	15q Tetrasomy 11.2-13.3
1	15q15.3 Deletion
1	15q15.4 Deletion
1	16p13.11 Deletion
1	16p13.11 Duplication; 17q12 Deletion
1	16p13.2 Duplication
1	16q22 Deletion
1	17p11 dup
1	17p13.3 Deletion
1	17q;21q Translocation

1	17q12 Duplication
1	17q21.31 Deletion
1	17q24.3q25.3 Duplication
1	18q Duplication
1	18q12.3 Deletion
1	18q21.1q21.32 Duplication
1	19q11-q13.11 Deletion
1	1p31.3 Duplication
1	1q24.2-1q25.3 deletion
1	1q41 Duplication
1	2 Duplication; 15 Duplication
1	2p16.3 Deletion
1	2q Deletion
1	2q duplication, hEDS
1	3p23q132 Inversion
1	3p25.3-3p26.3 Deletion
1	3q11 Deletion
1	3q26 Duplication
1	46,X,del(X)(q21.1)
1	46Xadd(Y)(q11.2)
1	4p Deletion Wolff-Hirschhorn Syndrome and 8p23.3 duplication
1	4p.16 Translocation
1	4q Deletion
1	4q26-q28.2 Deletion; 12p Deletion
1	4q34.3q35.2 Deletion
1	5p Deletion Cri du Chat; Trisomy 20p
1	5p14.1p14.3 Deletion
1	5q13.2 Deletion
1	5q14.3 Deletion
1	5q35 microduplication
1	6p Duplication; 6p Deletion
1	6p25.3 Deletion
1	6q Deletion
1	6q15-6q16.1 Deletion; 6q21 deletion
1	6q25.2-q26 Deletion
1	6q25.3 Deletion
1	6q25.3 Duplication
1	7p14 Deletion
1	7p22.1 Duplication
1	7p22.1p21.3 Deletion
1	7q Duplication
1	7q(del 7q34q35 and dup 7q35q36.1)
1	7q11.22 Deletion
1	7q11.23 duplication/2p21 deletion
1	7q11.2-q22.1 Duplication
1	7q31.1 Duplication
1	7q31.3q32.3 Deletion

1	7q32.1q32.2 Dup
1	8p Tetrasomy
1	8p11.21 Duplication
1	8p22p12 Deletion
1	8p22p21 Duplication
1	8p23.1 Duplication
1	8p23.2 Deletion; 8q24.22-q24.3 Duplication
1	8p23.2-8p23.1 triplication
1	8q22.1 Deletion
1	8q24.12 deletion
1	9p13.2 Duplication
1	9q21.12 9q21.13 Deletion
1	9q31.1-9q31.4 Deletion
1	ACAN
1	Acetabular Dysplasia
1	Achondroplasia; Down Syndrome
1	Achondroplasia; Geleo-physic Dysplasia
1	Acro-dermato-ungual-lacrimal-tooth Syndrome
1	Acromesomelic Dysplasia
1	AKT3 Megalencephaly polymicrogyria-polydactyly-hydrocephalus syndrome
1	Albright's hereditary osteodystrophy
1	Alpha-1-Antitrypsin Deficiency
1	Alstrom Syndrome
1	Arthrogryposis, Distal
1	Aural Atresia Unilateral
1	Auriculocondylar Syndrome
1	Axenfeld-Rieger
1	Baraitser-Winter syndrome
1	Bardet-Biedl; Moebius Syndrome
1	Beare-Stevenson Syndrome
1	Beta-Ketothiolase Deficiency
1	Beta-Propeller Protein-Associated Neurodegeneration
1	Biotinidase Deficiency
1	Bloom Syndrome
1	BPES Syndrome
1	Brachydactyly
1	Camurati-Engelann disease
1	Cantu Syndrome
1	Carnitine Deficiency; systemic primary
1	Cartilage Hair Hypoplasia
1	Catel-Manzke Syndrome
1	CDK13 Mutation
1	Cherubism Syndrome
1	Chromosome 6 intersitial deletion
1	Cleidocranial Dysplasia
1	Coffin Siris syndrome
1	Coffin-Siris Syndrome with Dandy Walker variant

1	COFS Syndrome
1	Congenital Adrenal Hyperplasia
1	Congenital Disorder of Glycosylation
1	Cranioectodermal Dysplasia
1	Craniometaphyseal Dysplasia
1	Cystic Fibrosis
1	D-bifunctional Protein Deficiency
1	Down Syndrome, Klinefelter
1	Duane retraction Syndrome
1	Dyskeratosis Congenita
1	Dystonia
1	Ear-patella-short stature Syndrome
1	Ectrodactyly
1	Emanuel Syndrome
1	Facioscapulohumeral Muscular Dystrophy
1	FBXO11 mutn
1	FG syndrome
1	Fibromuscular Dysplasia
1	Fibrous Dysplasia
1	Filippi Syndrome
1	Fucosidosis
1	Galactosemia
1	Gitelman Syndrome
1	Glutaric Acidemia
1	Gorlin Syndrome
1	Graves Disease
1	Greig cephalopolysyndactyly syndrome
1	Greig Syndrome
1	Gyrate Atrophy of Choroid and Retina
1	Hadju-Cheney syndrome
1	Helsmoorte-Van der Aa syndrome
1	Holoprosencephaly
1	Hypertelorism Microtia Facial Clefting Syndrome
1	Hypodontia
1	Hypokalemic periodic paralysis
1	Hypothalamic Pituitary Disorder
1	Hypotonia
1	IGF1R Resistance
1	Infantile Refsum Disease
1	isolated tracheoesophageal fistula
1	Kallmann Syndrome
1	Kartagener Syndrome
1	KAT6A Syndrome
1	Kearns-Sayre Syndrome
1	Kleeblattschädel Deletion Syndrome
1	Klinefelter Syndrome; Angelman Syndrome
1	Larsen Syndrome

1	Leri-Weill Dyschondrosteosis
1	Li-Fraumeni Syndrome
1	Limb-girdle muscular dystrophy
1	Malan syndrome
1	Marfan Syndrome, Neurofibromatosis type 1
1	Marshall Syndrome
1	Meckel and Joubert Syndrome
1	MECP2 triplication syndrome
1	MED13L
1	MED25
1	Megalencephaly-Capillary Malformation
1	Melnick-Needles Syndrome
1	Mental Retardation Autosomal Dominant 23
1	Mental Retardation, Autosomal Dominant 22
1	Metaphyseal acroscyphoDysplasia
1	Metaphyseal chondrodysplasia
1	Metatropic Dysplasia
1	Microophthalmia
1	Miller-Dieker Syndrome
1	Multiple Congenital Anomalies-Hypotonia-Seizures syndrome
1	Multiple Endocrine Neoplasia
1	Multiple Epiphysial Dysplasia
1	mutation in KDMSC gene
1	Myotonic Dystrophy
1	Neurocardiogenic Synchopy
1	Oligodontia
1	Orofacial digital Syndrome NOS
1	Orofaciodigital Syndrome
1	PBX1 Variant
1	PDCH19
1	Potocki Shaffer Syndrome
1	Proprionic Acidemia
1	Pseudohypoparathyroidism
1	Pseudoxanthoma Elasticum
1	Recombinant 8 Syndrome
1	Rodriguez
1	Schimke immuno-osseous Dysplasia
1	Scleroderma
1	Seckel Syndrome
1	SEDc vs Kniest - two Orpha #s
1	Setleis Syndrome
1	Shashi-Pena Syndrome
1	Short chain acyl-CoA dehydrogenase deficiency
1	Shprintzen-Goldberg craniosynostosis Syndrome
1	Situs Inversus
1	Spondyloenchondrodysplasia with Immune Dysregulation
1	Spondyloepimetaphyseal Dysplasia; Stickler Syndrome

1	SSR4
1	Syndactyly 3/4 fingers, polydactyly toes
1	Tatton-Brown-Rahman Syndrome
1	Temple Syndrome
1	Tessier cleft
1	Trisomy 9 mosaicism
1	Trisomy 9p
1	TUBA1A-related disorder
1	UPD 14
1	Van Maldergem syndrome 2
1	Very-long-chain acyl-CoA dehydrogenase deficiency
1	Williams-Beuren Syndrome; Marfan Syndrome
1	X-Linked Hypophosphatemic Rickets
1	X-linked Ichthyosis
1	X-linked mutation of AMER1
1	Xp11.4 deletion
1	Xp22.11p11.4 Deletion; SRY+; 5p13.3 gain
1	Xp22.31 Deletion
1	Xp22.33 Deletion/EDS type 2
1	Xp22.33p22.32x3
1	Xq13.1 Duplication
1	Xq21.1 Deletion
1	Xq22.1 Deletion
1	Xq22.3-q28 Deletion
1	Xq25 Duplication
1	Xq28 deletion syndrome, 4p partial trisomy syndrome
1	XY/XO mosaicism
1	Y Chromosome anomaly
1	You-Hoover-Fong Syndrome
1	Yp11.2-Yq11.23 Duplication
