# Supplementary material for: Automated syndrome diagnosis by three-dimensional facial imaging
Source: Genet Med. 2020 Jun 1;22(10):1682–93. doi: 10.1038/s41436-020-0845-y (PMC7521994; doi:10.1038/s41436-020-0845-y)
Supplement: Supplementary file 3 — Supplementary File S1 [file 41436_2020_845_MOESM3_ESM.pdf]

Code

Sampling issues with holdout cross-validation

Any holdout sampling done when syndrome sample sizes are small will almost certainly poorly represent the underlying distribution. This simple example shows why with a univariate example where we've held out 30% of observations. Holdout sets on small sample sizes are unstable, and will tend to be biased to the mean, ignoring the distinctive characteristics of syndromes where they exist. This effect is exacerbated as you add dimensionality.

Red is underlying true distribution, blue ticks are sampled holdout distribution of 30% of the data. When data is plentiful, the sampling distribution approximates the true distribution. When data is sparse, like with genetic syndromes, the sample can not approximate the true underlying distribution. Thus, any inference from small samples based on holdout validation strategies are meaningless.

Hide

```
set.seed(587)
holdout.perc <- .30

vision <- rnorm(20000)
vsample <- sample(vision, size = holdout.perc * length(vision), replace = F)

# pdf(file = "/mnt/Hallgrimsson/Users/Jovid/FB2_ML/figures/largeN_holdout.pdf", width = 8, heigh
t = 5)
plot(density(vision), typ = "n", main = "Large sample (n = 20k) holdout validation set", xlab =
"x")
polygon(density(vision), col = rgb(1,0,0, alpha = .5))
```

Hide

```
points(rep(.07, length(vsample)) ~ vsample, col = rgb(0,0,1, alpha = .5), type = "h", lwd = 1.5)
```

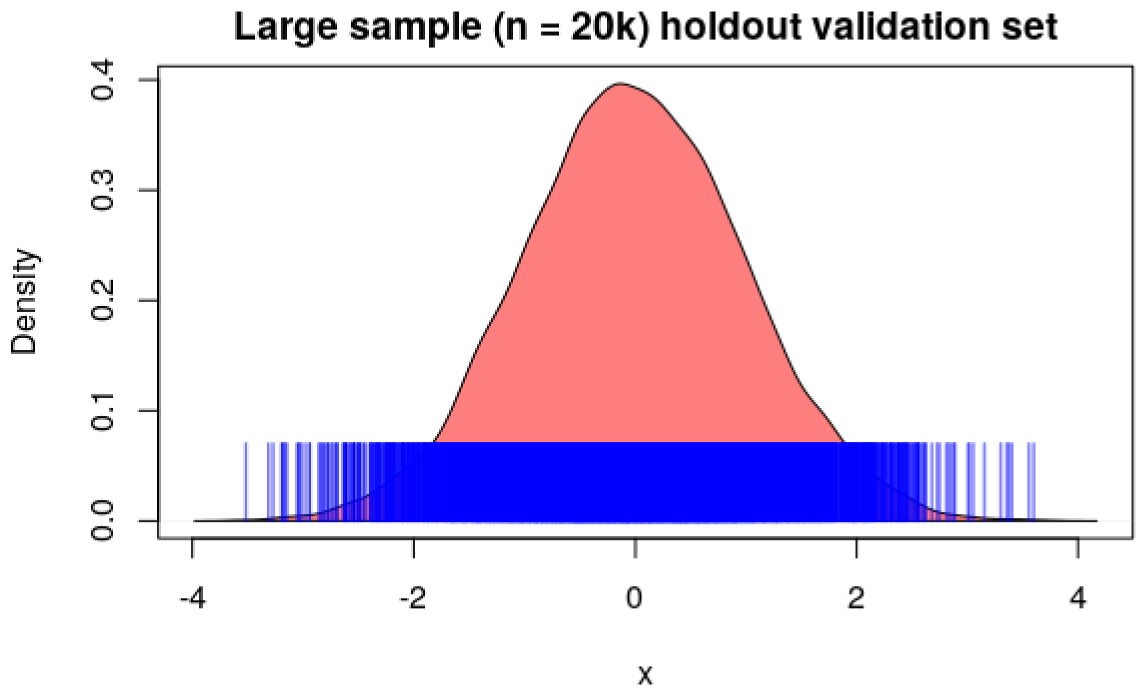

Hide

```
# dev.off()

mlsyndrome <- rnorm(10)
msample <- sample(mlsyndrome, size = holdout.perc * length(mlsyndrome), replace = F)

# pdf(file = "/mnt/Hallgrimsson/Users/Jovid/FB2_ML/figures/smallN_holdout.pdf", width = 8, heigh
t = 5)
plot(density(mlsyndrome), typ = "n", main = "Small sample (n = 10) holdout validation set", xlab
= "x")
polygon(density(mlsyndrome), col = rgb(1,0,0, alpha = .5))
```

Hide

```
points(rep(.07, length(msample)) ~ msample, col = rgb(0,0,1, alpha = .5), type = "h", lwd = 1.5)
```

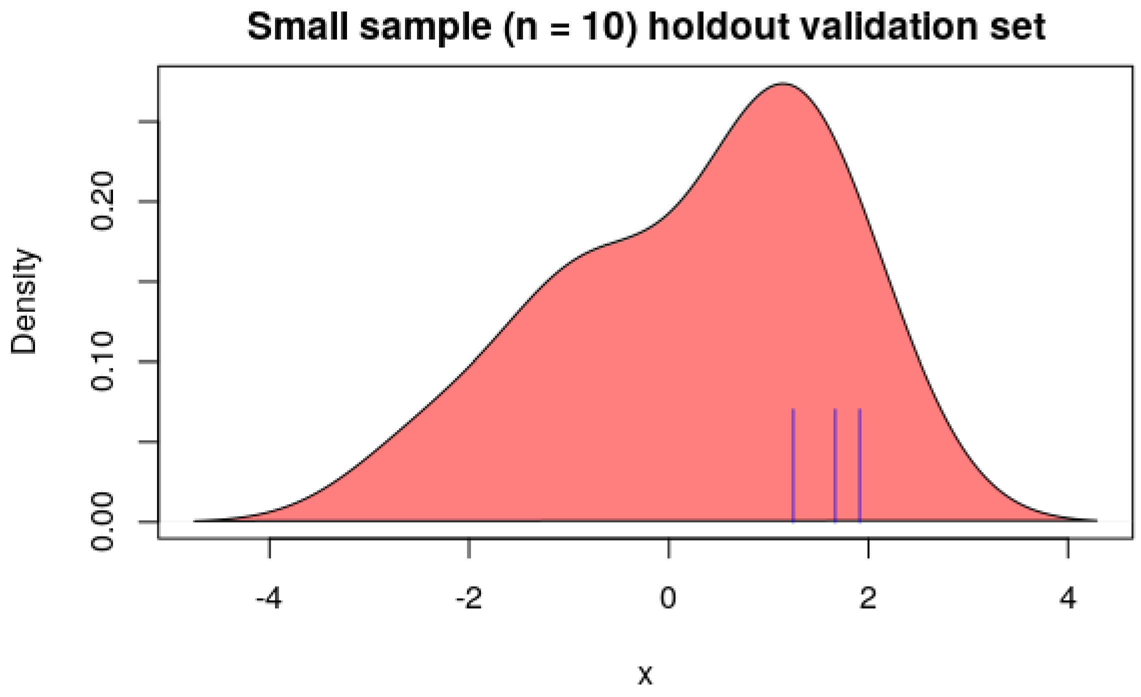

Hide

```
# dev.off()
```

Now let’s imagine trying to discriminate between two syndromes with real differences under well-conditioned and poorly conditioned holdout strategies.

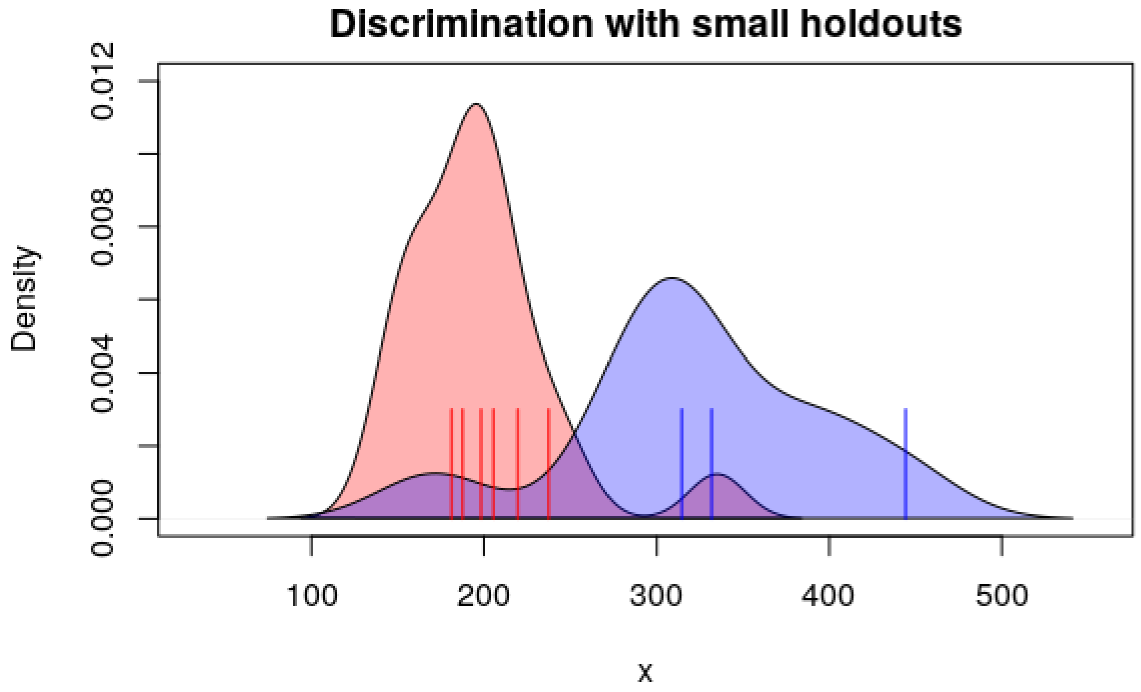

You can see that the estimate of discrimination accuracy will be highly unstable, and will more often reflect sampling artifacts than true differences. With a 75% difference in mean, we expect at best 75% (expected sensitivity will change with differences in variance as well), but here we’d report 100% sensitivity for the blue group and 83% sensitivity for the red group.
