# Supplementary material for: Automated syndrome diagnosis by three-dimensional facial imaging
Source: Genet Med. 2020 Jun 1;22(10):1682–93. doi: 10.1038/s41436-020-0845-y (PMC7521994; doi:10.1038/s41436-020-0845-y)
Supplement: Supplementary file 4 — Supplementary File S2 [file 41436_2020_845_MOESM4_ESM.pdf]

File S3: Vector maps for syndromes arranged along among-syndrome PC

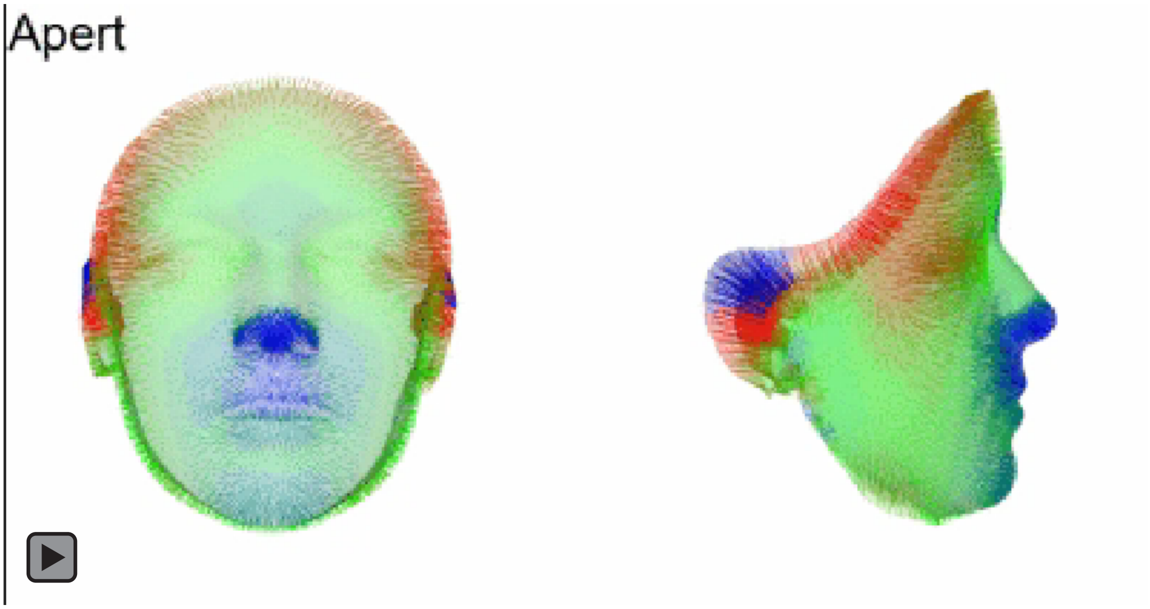

PC1 (click on image to animate)

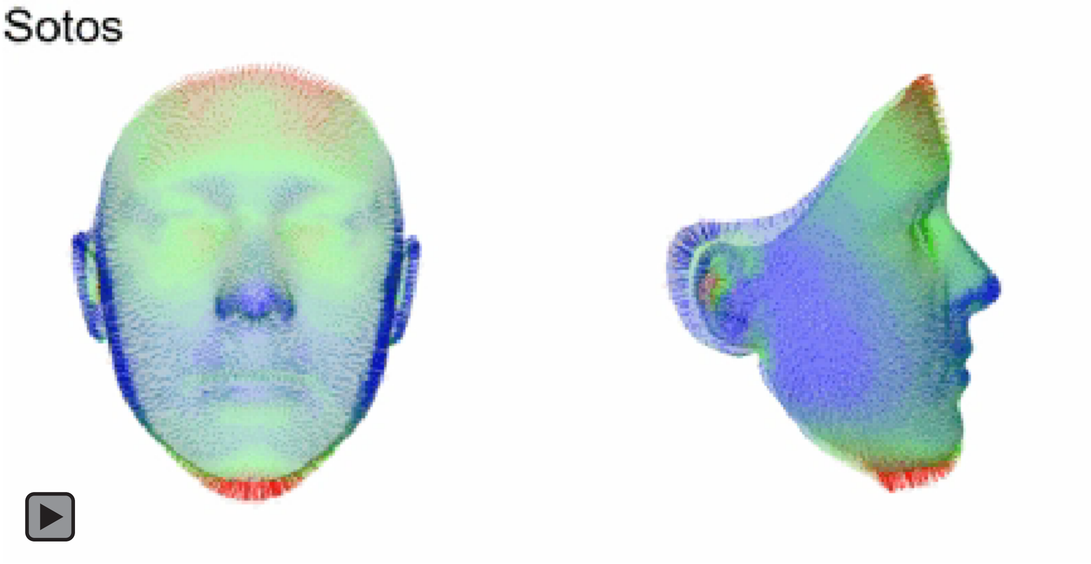

PC2 (click on image to animate)

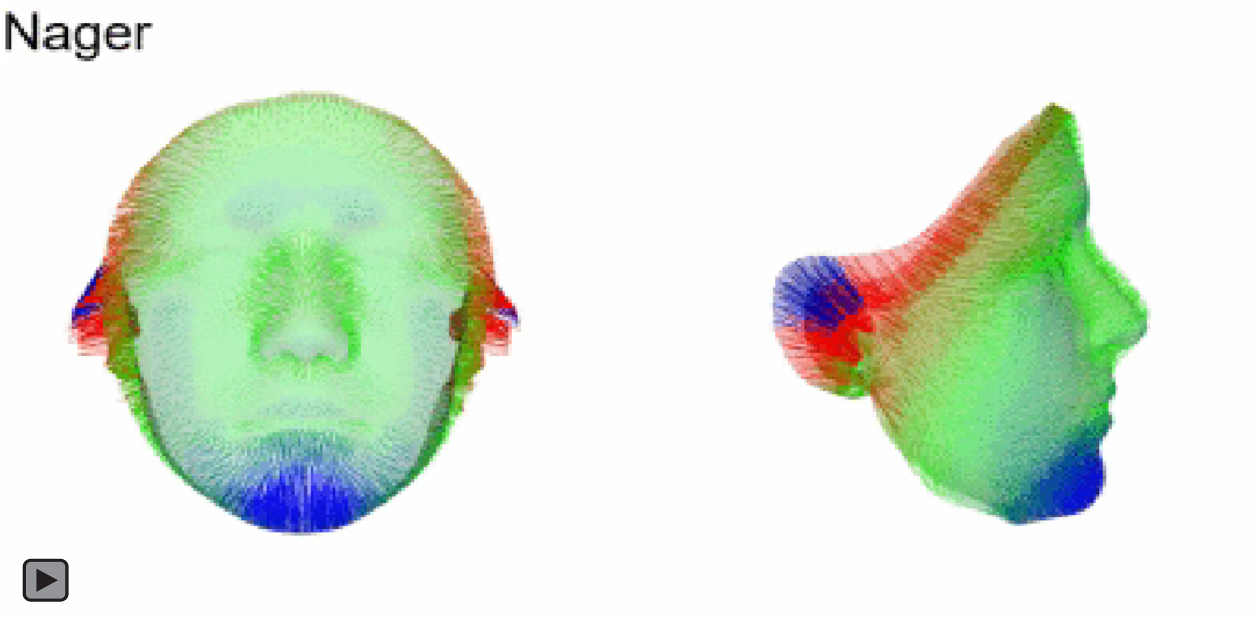

PC3 (click on image to animate)

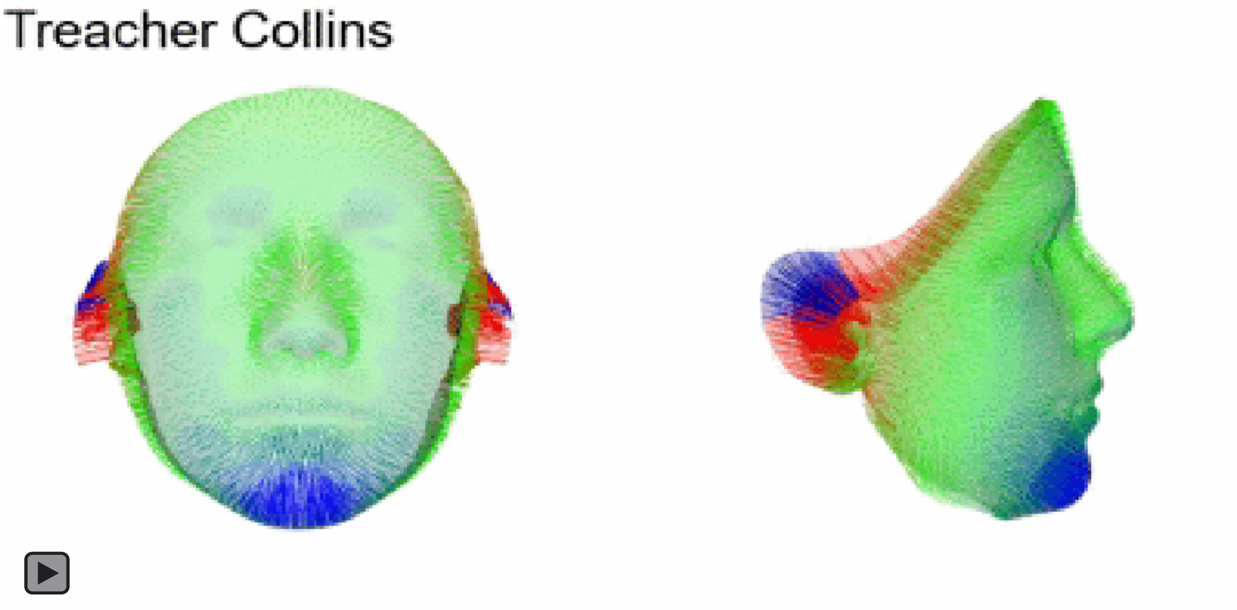

PC4 (click on image to animate

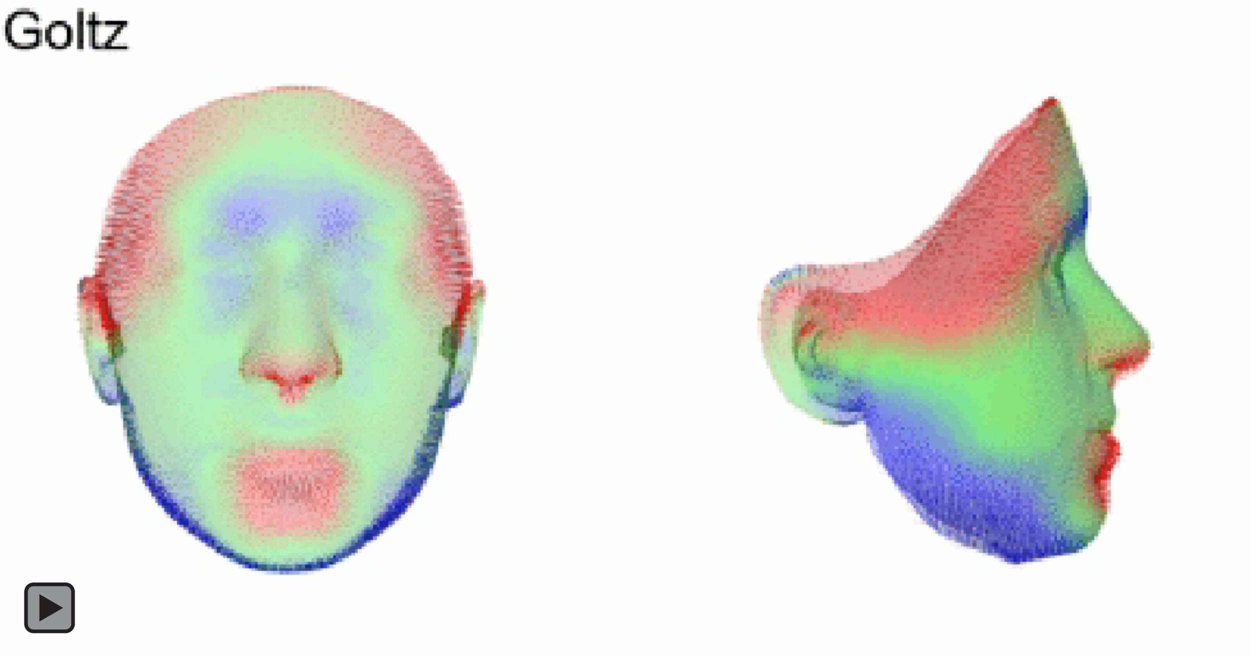

PC5 (click on image to animate)

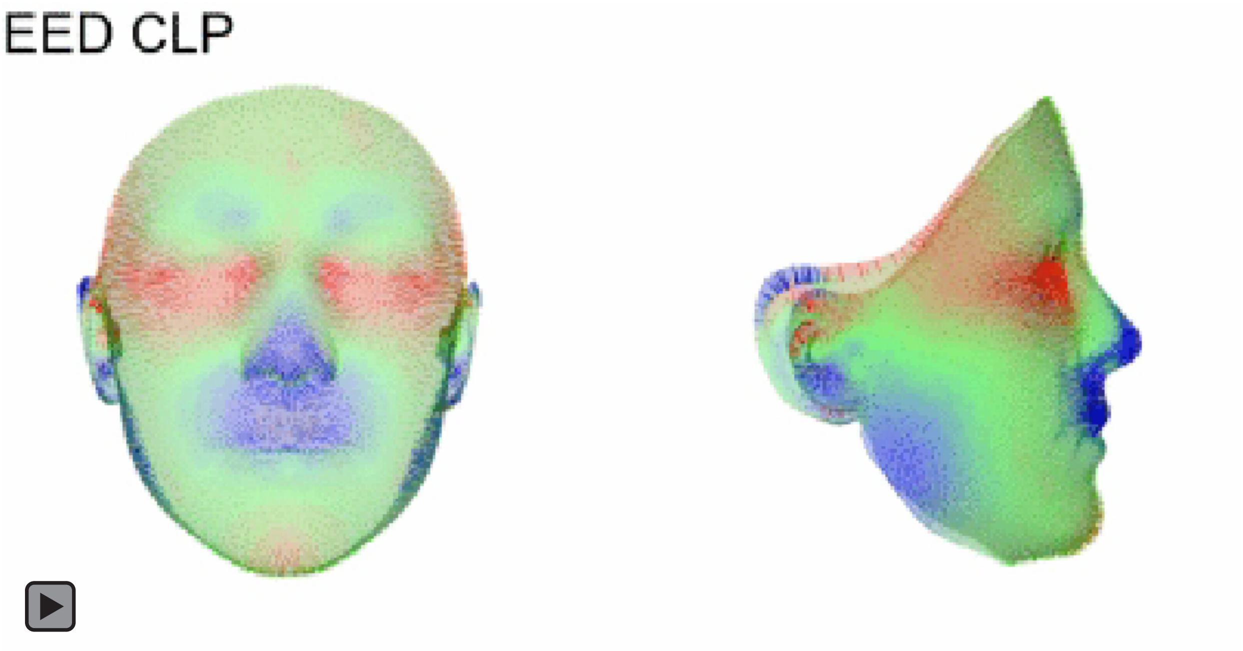

PC6 (click on image to animate

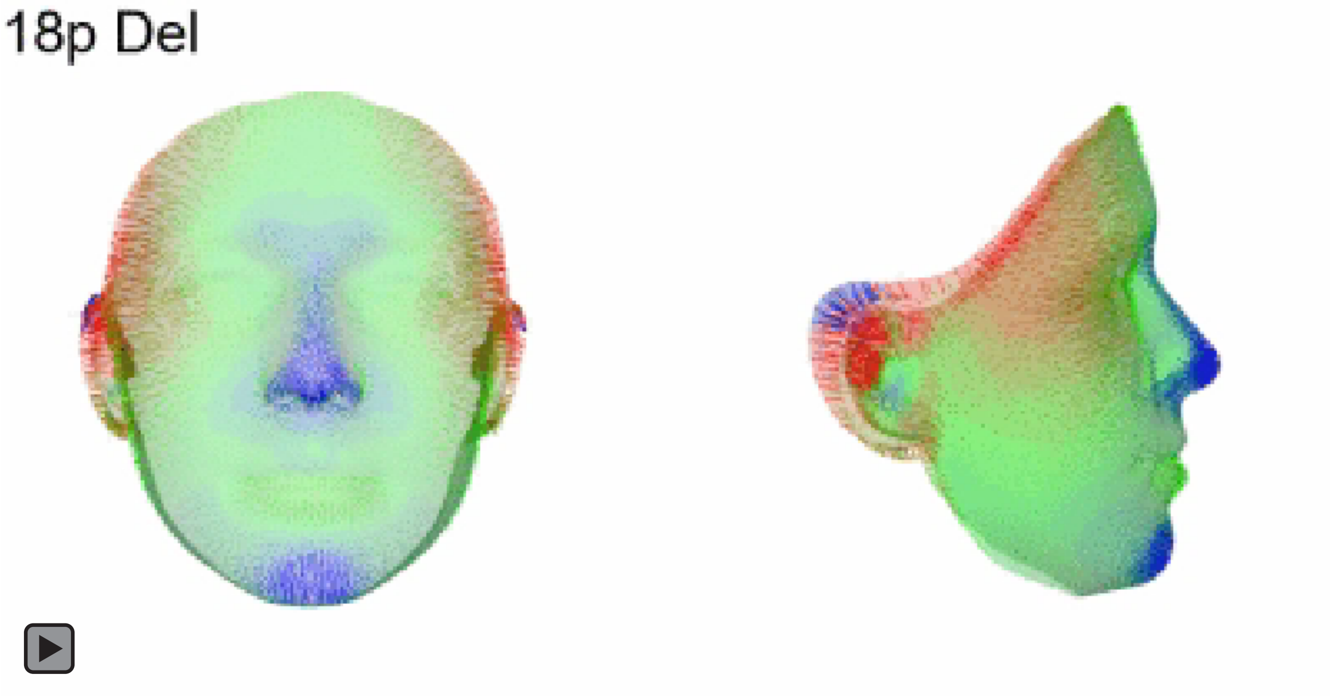

PC7 (click on image to animate)

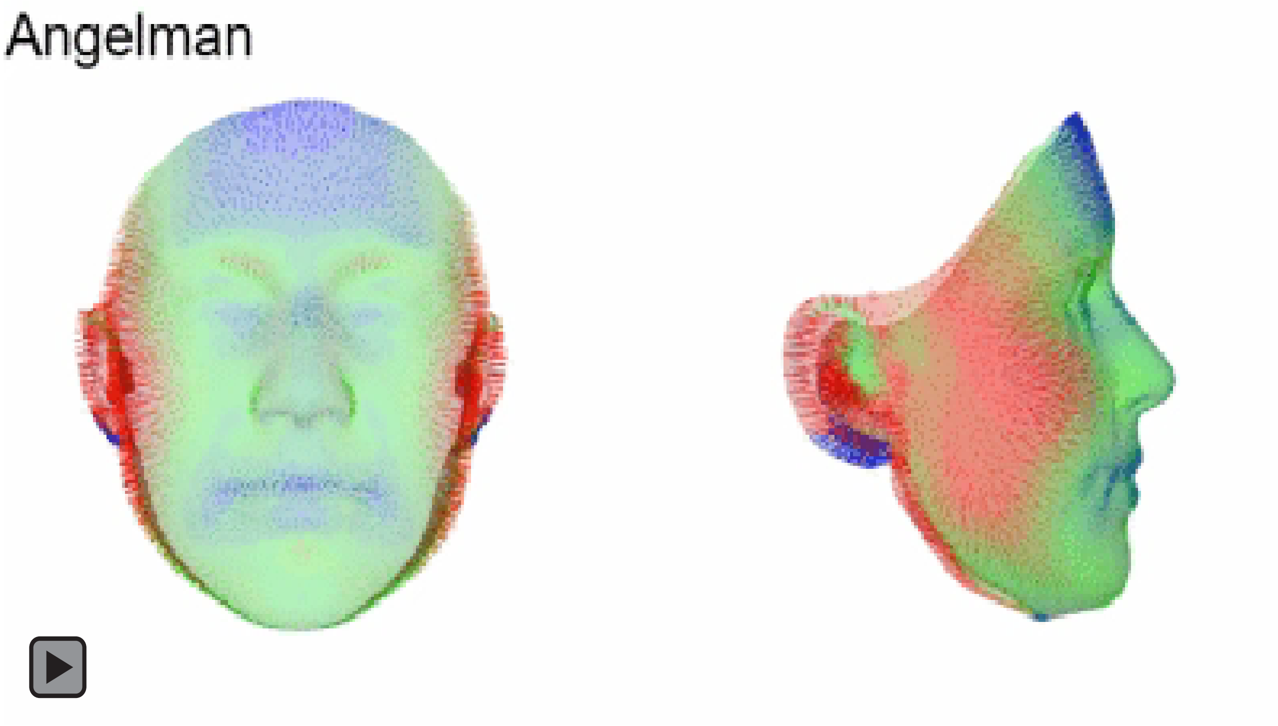

PC8 (click on image to animate)
